# CSF in the ventricles of the brain behaves as a relay medium for arteriovenous pulse wave phase coupling

**DOI:** 10.1371/journal.pone.0181025

**Published:** 2017-11-15

**Authors:** William E. Butler, Pankaj K. Agarwalla, Patrick Codd

**Affiliations:** 1 Massachusetts General Hospital, Neurosurgical Service, Boston, Massachusetts 02114, United States of America; 2 Duke University Medical Center, Department of Neurosurgery, Durham, North Carolina 27710, United States of America; Ehime University Graduate School of Medicine, JAPAN

## Abstract

The ventricles of the brain remain perhaps the largest anatomic structure in the human body without established primary purpose, even though their existence has been known at least since described by Aristotle. We hypothesize that the ventricles help match a stroke volume of arterial blood that arrives into the rigid cranium with an equivalent volume of ejected venous blood by spatially configuring cerebrospinal fluid (CSF) to act as a low viscosity relay medium for arteriovenous pulse wave (PW) phase coupling. We probe the hypothesis by comparing the spatiotemporal behavior of vascular PW about the ventricular surfaces in piglets to internal observations of ventricle wall motions and adjacent CSF pressure variations in humans. With wavelet brain angiography data obtained from piglets, we map the travel relative to brain pulse motion of arterial and venous PWs over the ventricle surfaces. We find that arterial PWs differ in CF phase from venous PWs over the surfaces of the ventricles consistent with arteriovenous PW phase coupling. We find a spatiotemporal difference in vascular PW phase between the ventral and dorsal ventricular surfaces, with the PWs arriving slightly sooner to the ventral surfaces. In humans undergoing neuroendoscopic surgery for hydrocephalus, we measure directly ventricle wall motions and the adjacent internal CSF pressure variations. We find that CSF pressure peaks slightly earlier in the ventral Third Ventricle than the dorsal Lateral Ventricle. When matched anatomically, the peri-ventricular vascular PW phase distribution in piglets complements the endo-ventricular CSF PW phase distribution in humans. This is consistent with a role for the ventricles in arteriovenous PW coupling and may add a framework for understanding hydrocephalus and other disturbances of intracranial pressure.

## Introduction

The arrival to the brain in the rigid cranium of a stroke volume of arterial blood must be matched over the cardiac cycle by an equivalent volume of exiting venous blood [1]. The kinetic energy of entering arterial blood must be matched over the cardiac cycle after frictional losses by the kinetic energy of exiting venous blood.

A related article introduces a method for wavelet brain angiography and shows it to resolve individual vascular pulse waves (PWs) in the brain from high speed angiographic data while simultaneously measuring extravascular brain pulse motion [2]. That method exploits angiographic time of flight (ATOF) to separate arterial from venous circulation and finds that circulation in the brain contains distinct but phase locked arterial and venous PWs that each maintain separate phase relations to brain pulse motion. Those findings are consistent with there being a PW mediated information exchange between the arterial and venous circulations as might be needed to conserve a balance of blood volume and cardiac kinetic energy about the cardiac cycle between the arterial and venous circulations.

If arterial PWs are reciprocally coupled to venous PWs by the pulse motion of brain tissue, might the stiffness and viscosity of brain tissue limit the coupling?

The ventricles of the brain are cerebrospinal fluid (CSF)-filled chambers that span the cranial cavity. They remain the largest anatomic structure in the human body without established primary purpose, even though their existence has been known at least since described by Aristotle [3]. This ignorance limits progress against hydrocephalus and other disturbances of intracranial pressure. If CSF in the ventricles were to serve as a low viscosity PW relay medium, it might enhance the efficiency of vascular PW coupling by providing a low viscosity medium for pulse motion conduction that spans the compartments of the the cranial cavity. We report here an exploratory application of wavelet brain angiography to the question of whether CSF as configured by the brain’s ventricles displays spatial and timing properties consistent with functioning as a relay medium for arteriovenous PW coupling.

This hypothesis predicts that: (1) arterial and venous PWs over the surfaces of the ventricles have separate phase relations to brain pulse motion; (2) the phase of a traveling vascular PW has a consistent spatial distribution over the surfaces of the ventricles; (3) seen from within, the ventricle walls move periodically at cardiac frequency (CF) with a consistent spatial phase distribution; (4) CSF pressure within the ventricles varies at CF with a consistent spatial phase distribution; and (5) these vascular and CSF phase distributions share spatiotemporal correspondences.

We test prediction (1) by comparing the phase relative to brain pulse motion of arterial and venous PWs over the surfaces of the ventricles in wavelet brain angiography data from piglets [2]. We test prediction (2) by mapping in those data the spatial distribution of vascular PWs phase over the surface of the ventricles. We test predictions (3) and (4) with data gathered directly from within human brain ventricles during endoscopic third ventriculostomy (ETV), a clinical procedure where an endoscope capable of recording optical video and gathering CSF pressure is positioned within the ventricles. We test prediction (5) by comparing the angular distributions of piglet peri-ventricular and human intra-ventricular PW phase data.

The morphological matching in this paper of piglet and human timing data rests on the physiological assumption that cardiac cycle phenomena in the brain in humans and piglets both occur with respect to the cardiac cycle rather than to absolute timing. Expressing timing data in radians with normalization by the cardiac period gives mathematical correspondence to the complex-valued wavelet methods that are employed in this paper. A complex number *a* + *ib* can be expressed in polar notation as magnitude given by $\sqrt{a^{2}+b^{2}}$ and phase *ϕ* given by the angle between the origin and the vectors {1, 0} and {*a*, *b*}. If two complex-valued numbers *x* and *y* represent CF data, their phase difference is the angle in polar notation of *x* multiplied by the complex conjugate of y*, *ϕ*(xy*), where the superscript * denotes complex conjugation.

## Materials and methods

### Piglet cranial window ultrasound angiography

The related article describes the piglet cranial window ultrasound angiography data and the wavelet angiography methods [2]. That study was approved by the institutional Subcommittee on Research Animal Care, and the experiments were performed in three piglets (designated p1, p2 and p3). We provide here as Supporting Information the raw angiographic data and their wavelet cine PW reconstructions that are subjected to further analysis in this paper. Briefly, under the care of a veterinarian, the piglets were premedicated with intramuscular xylozine 2 mg/kg then general anesthesia was induced with an intramuscular tiletamine/zolazepam combination and maintained with inhalational isoflurane. A craniectomy at the coronal suture of the cranium is performed, with the bone removal size corresponding to the head of an ultrasound probe. The probe is rigidly mounted, then a bolus of perflutren, an ultrasound contrast agent (Definity, Lantheus Medical Imaging, North Billerica, Massachusetts), is briskly administered while ultrasound cine is recorded at a sampling rate significantly higher than CF [4]. The ultrasound imaging is with a linear array probe at 8.3 MHz (MySonos 201, Medison). The animals were maintained under general anesthesia throughout, then euthanized while still under anesthesia with an intravenous pentobarbital overdose.

### Wavelet angiography

Per the wavelet angiography technique described in the related article, the cine ultrasound video frames that capture the passage of the angiographic bolus are reorganized in computer memory such that each pixel represents a time signal corresponding to its spatial location [2]. The angiographic time signal at the *i*,*j*^*th*^ pixel is denoted *c*_*i*,*j*_ and the overall angiographic signal is denoted *C*. Sampling by uniformly spaced time is implied. The over hat symbol ^ signifies a high temporal resolution wavelet transformation and the over tilde symbol $\;\tilde{}\;$ signifies a high frequency resolution wavelet transformation. The high temporal resolution wavelet transform serves to mitigate pulse motion alias and the cross-correlated high frequency resolution wavelet transform mitigates frequency alias. As mentioned above, the wavelet PW reconstructions are provided as Supporting Information video files.

The angiographic CF phenomena are extracted by calculating

$$\begin{array}{c} {\hat{x}}_{i,j}={\hat{c}}_{i,j}\;{\tilde{C}}^{*} \end{array}$$ (1)

followed by filtering ${\hat{x}}_{i,j}$ for cardiac wavelet scale at inverse wavelet transformation (S1 File). This gives a spatiotemporal grid where each grid element has a complex-valued datum that represents its CF angiographic state. Cine images of the spatiotemporal CF decomposition can be rendered with a brightness-hue color model, where the magnitude of a complex-valued datum is represented as brightness and phase as hue.

The wavelet angiography code is written using the Wolfram language in the Mathematica version 11 (Wolfram Research, Champagne, Illinois, USA) environment. In that product’s library, the high frequency and high temporal resolution wavelet transforms are reified by the GaborWavelet functions respectively with parameter values 6 and 1.

#### Brain pulse motion

Brain pulse motion is quantified by tracking the frame-wise displacements of a single object in the image and then reducing the 2-D displacements to a one-dimensional signal, *M*, as described in the related article [2]. The object to be tracked is selected opportunistically in the video footage as that with the clearest motion to visual inspection. These methods do not have the resolution to track distributed motions throughout a complex structure such as the ventricle walls of the piglet. The same method and computer source code are employed for measuring human brain ventricle wall motion from neuro-endoscopic video as below.

The related article gives the modification of Eq 1 to give wavelet angiography by reference to brain pulse motion,

$$\begin{array}{c} \hat{y}=\hat{c}{\left(\tilde{C}{\tilde{M}}^{*}\right)}^{*}=\hat{c}\;{\tilde{C}}^{*}\tilde{M}. \end{array}$$ (2)

Brain pulse motion serves as a local chronometer to compare the vascular PW phase early in angiographic flight to that late in angiographic flight. Brain pulse motion accordingly serves as the reference for comparing arterial and venous PW phase.

#### Peri-ventricular vascular pulse wave distribution with temporal profile

**Fig 1:**
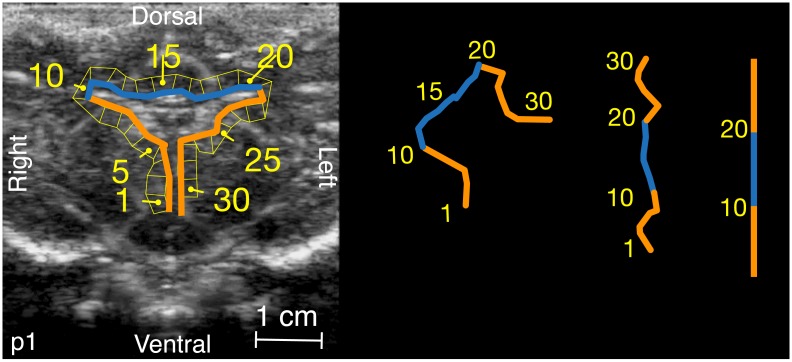
Peri-ventricular polygons. The orientation and segment lengths of the inner line are preserved during the rotation and unwinding.

Employing the data reported in the related article, we trace a line over the outer surface of the Lateral and Third Ventricles on the ultrasound images of each piglet, with guidance from a piglet brain atlas and from a brain magnetic resonance image in one of the animals [2], [5]. The line is divided into segments number 1 to 30 of equal length. This segmented line is used to generate by an automated method an array of contiguous quadrilateral polygons spanning the outer surface of the Lateral and Third Ventricles (Fig 1). The equal lengths of the inner segments of the polygons preserve a one-to-one correspondence between the ventricular perimeter on the anatomic drawing and the vertical axis on the pulse incidence plots (Fig 1).

With the passage of an intravenous contrast bolus of perflutren, the mean of the complex-valued CF angiographic phenomena in each polygon is computed with time indexing. The areas in the polygons are not equal, but the mean rather than the sum complex valued CF data are obtained from each. The cranial window ultrasound sampling of the angiographic bolus was sampled for p1 at 30 Hz for 21.7 seconds, for p2 at 30 Hz for 11.3 seconds, and for p3 at 10 Hz for 14.0 seconds. This gives a spatial array of CF complex-valued time signals which can then be unfolded for graphical display. Multiplying the array size of 30 polygons by the sampling rate by duration gives the size of the complex-valued data table analyzed for each piglet. The segments forming the ventral walls of the Third and Lateral Ventricles are depicted in orange and the segments for the dorsal segment of the Lateral Ventricle in turquoise (Fig 1). The mean value of magnitude is rendered as brightness and phase as hue. These values depict sequences of individual vascular PWs about an unfolded surface profile of the ventricles.

#### Peri-ventricular arterial versus venous pulse wave phase difference

The peri-ventricular angiographic time intensity curve of a wavelet angiogram is inspected and arterial and venous time intervals of about 5 heart beats in length are selected before and after the time point of peak angiographic intensity. The complex-valued data about the peri-ventricular zone with respect to brain pulse motion are averaged across these two time intervals, and the phase is taken to represent respectively the peri-ventricular arterial and venous PW phases. The means of these phase distributions are compared by Watson’s two-sample test for circular data [6].

#### Dorsal-ventral pulse wave relationship

To describe the trend of dorsal versus ventral peri-ventricular surface vascular PW magnitude and phase, the complex-valued time domain signals from the dorsal and ventral walls are averaged separately after referencing for brain pulse motion phase to give two complex-valued signals, *V*_*Dorsal*_ and *V*_*Ventral*_, where time indexing is implied. In the peri-ventricular polygons, the ventral surface polygons are sequence numbers 1-10 and 21-30 colored in orange in Fig 1, and the dorsal surface polygons are 11-20, colored in turquoise. For this analysis the data table size for each piglet is two times the sampling rate by duration. Since *V*_*Dorsal*_ and *V*_*Ventral*_ are products of a wavelet CF filter, their phase difference can be calculated as the argument (angle in the complex plane) of $V_{Dorsal}{V_{Ventral}}^{*}$, where the superscript * represents complex conjugation. The dorsal and ventral faces are selected for analysis because the PW data shown in unfolded vascular PW profiles suggests this to be a natural boundary in PW activity. The mean of the phase differences is compared to zero radians by Watson’s non parametric test for circular data [7].

### Neuro-Endoscopy

#### Subjects

**Fig 2:**
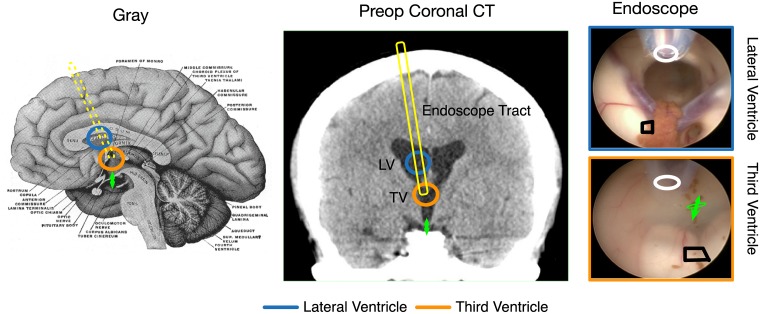
Lateral and third ventricle orientation in human endoscopic third ventriculostomy. Annotated drawing from Gray and Carter (left), coronal CT from h1 (middle), and endoscope views within the Lateral and Third Ventricles (right). The approximate Lateral Ventricle position is in turquoise, Third Ventricle position in orange, endoscope tract is in yellow, and the green arrow gives the therapeutic fenestration site. In the endoscope views the black polygons give the motion tracking region and the white ovals give the catheter tip positions for CSF pressure transduction.

Selected human patients with hydrocephalus may undergo attempted treatment by ETV. In this procedure, an endoscope is placed transcortically into the Lateral Ventricle, advanced through the foramen of Monro into the the Third Ventricle, and a fenestration is made at the floor of the Third Ventricle to provide an avenue of egress of CSF from the ventricles to the subarachnoid space, where it can then reabsorbed (Fig 2) [8]. The Lateral Ventricles are anatomically positioned in dorsal relation to the Third Ventricle. With approval by Partners Human Research Committee and approached six subjects for informed written consent in the interval between October 2014 and April 2015. We completed the acquisition of direct ventricle wall motion and adjacent CSF pressure data in three (designated h1-h3). The three subjects who signed consent but for whom at surgery we did not gather data are described further below.

#### Operative procedure

We pause the procedure for approximately 10 heartbeats when the endoscope tip is in the Lateral Ventricle and in the Third Ventricle, in each case before and after the fenestration of the Third Ventricle floor. In granting approval, the Partners Human Research Committee ruled that acquiring ten heartbeats of data at each of four stages in the procedure would not extend the duration of the procedure so as significantly to add risk or discomfort of the subjects.

We adhere to our neurosurgical routine for this procedure which is for it to be performed cooperatively by two surgeons. One holds the endoscope with two hands while it is in the ventricles of the brain while through the working channels the other manipulates the irrigation catheter and passes the spreading forceps to fenestrate the floor of the Third Ventricle. There is continuous video recording throughout the procedure. For a subject enrolled in the study, the alteration in routine is that one surgeon holds the endoscope while the other locks it in place with a rigid endoscope holder that employs a pneumatic passive brake (Mitaka UniArm, Mitaka USA, Park City, Utah, USA). During the 10 heartbeat interval a three way stopcock on the irrigation catheter is turned to connect the fluid column to a pressure transducer, temporarily turning the irrigation catheter into a pressure conduit. This permits with minimal alteration in surgical routine the simultaneous recording of CSF pressure variations and video. The location of the endoscope tip where the measurements are made is determined by clinical judgment during each procedure and is therefore not a parameter that can be tightly controlled between subjects including not in a manner to compensate for the differences in ventricular morphology between these humans and the piglets. Regardless of variabilities in endoscope positioning, in each case the position of the endoscope in the Lateral Ventricle is dorsally located relative to its position in the Third Ventricle. The positions of the endoscope tip may be gathered from the recorded video position (shown in Fig 2 for h1, S1 Fig for h2, and S2 Fig for h3). Because of these imprecisions, this paper emphasizes graphical analysis over inferential statistics.

Three other subjects signed consent but physiological data were not gathered during their surgery because anomalies were encountered. These anomalies could have been corrected during the procedure to enable the planned data collection but this would have called for one surgeon manually to hold the endoscope atraumatically in the brain for a period of time as the other addressed the anomaly. We judged in each case that the added risk and operative time did not justify pausing the procedure so we aborted the scientific component of the procedure and instead completed the clinical goal. In one, the pressure transduction catheter had a bubble that did not flush with gentle force. We elected not to pull it out of the brain to flush it forcefully and reinsert it. In another, the endoscope holding arm wobbled and we elected not to pause to retighten its attachment to the operating table. In the third, the synchronizing “finger flick” described below was not captured initially in the video recording and we elected not to pull the endoscope out of the brain to acquire it. We do not assess as likely that these subjects where data was not captured to have differed significantly from the those in whom it was.

In this paper, we analyze only the data obtained before the fenestration because the aim of this study is to shed light on ventricle function, not to study the effect of ETV. The impact of the Third Ventricle fenestration on brain ventricle physiology merits separate study. The CSF pressure waveform is gathered by a standard fluid-coupled pressure transducer (Truwave Disposable Pressure Transducer, Edwards Lifesciences, Irvine, California, USA) attached to a general anesthesia machine (Apollo Anesthesia Machine, Drager Medical Inc., Telford, Pennsylvania, USA). As the endoscope is manipulated throughout the procedure, the catheter position at the endoscope tip is in motion, but the atmosphere reference transducer remains in fixed position outside of the sterile field. The waveform is digitized and stored (Masimo SET, Masimo Corporation, Irvine, California, USA) along with manometric blood pressure and ECG waveforms in 32 bit integer format for subsequent analysis. At analysis, blood pressure and ECG waveforms provide the timing reference for synchronized analysis of the Lateral and Third Ventricle data.

The video is recorded by a video system at 30 Hz, and is downloaded from a video recording device in the operating room as an video file (either in .avi or in .mov format, depending on the video recording device brand that is present). The metadata of the video files report 3000 kb/s with 1280 by 720 pixel resolution in red, green, blue color format at 8 bits per color channel. For analysis, the area of interest is extracted and the frames are converted to 8 bit grayscale format using the video conversion software tool ffmpeg (version 2.6.1, available from http://ffmpeg.org). The endoscope video camera is a wide angle lens, so the spatial scale in the image depends on distance of a given object from the camera, but it can be estimated from the 5 French suction catheter as seen in the figures and video files since by convention 3 French scales to 1 mm.

The video footage and the CSF pressure waveforms are gathered by separate, unintegrated instruments in the operating room, and therefore are not synchronized at the time of acquisition. In order to synchronize them the pressure-transduction catheter is physically placed directly in the endoscope field of video view and the catheter is sharply displaced (“finger flicked”) so as to produce a distinct pressure waveform and video-recorded motion pair that can be used post-hoc to synchronize the endoscopy video and CSF pressure traces (see S3 Fig). We wrote a software tool with a graphical user interface to perform the alignment, and attach as Supporting Information a video file recording an example of its usage (S1 Video). We estimate this technique to offer synchronization to within about one video frame.

#### Human ventricle wall speed

The speed of ventricular wall motion is measured from the video by tracking the two dimensional displacements of a distinctly visible object with the same computer code as used to track brain pulse motion in the piglet cine ultrasound data [2]. Typically, the motion per frame is less than 1 pixel, but by including hundreds of pixels in a given region of interest, a net displacement of less than 1 pixel per frame can be measured. As with the analysis of piglet brain pulse motion, the two dimensional displacement data are projected along the major eigenvector axis of their covariance matrix to give one-dimensional ventricle wall speed. We do not examine the physiological significance of the directionality of the wall motion vectors.

The positions of the CSF pressure transduction catheter and the motion tracking regions of interest for human subject h1 are depicted by the black polygons in the right column of Fig 2. Similar figures for h2 and h3 are S1 and S2 Figs.

#### Wavelet analysis of human cardiac frequency CSF phenomena

The CSF pressure signals are each wavelet transformed with the GaborWavelet [7] function offered by the Mathematica environment. Then, after cardiac cycle alignment using the plethysmographic blood pressure or ECG signal, the phase difference between the Lateral and Third Ventricle waveforms is obtained using a cross-wavelet method [9–12]. The phases of the cross-wavelet represent the phase differences between the two Lateral and Third Ventricle CSF pressure signals. These angular differences of dorsal minus ventral CSF pressure CF phase are gathered into circular histograms for display. The mean of the phase differences is compared to zero radians by Watson’s non parametric test for circular data [7].

### Cardiac frequency phase statistics

The mean and standard deviation of CF phase is computed with circular statistics methods since angular space is non-Euclidean [13]. Cross-correlated wavelets yield measurements of dorsal minus ventral CF phase differences in piglet peri-ventricular vascular PW phase and in human endo-ventricular CSF PW phase [9–12]. The distributions of the phase differences are separately inspected directly as circular histograms. Tests for the mean of a distribution of CF phase differences being similar to zero radians are by Watson’s non parametric test for circular data [7]. For comparison, their empirical angular distributions are matched by quantile to give Q-Q plots [14]. In this exploratory analysis we deemphasize inferential statistics in favor of graphical analyses.

## Results

### Piglet ventricle surface CF angiographic phenomena

#### Dorsal-ventral PW relation

**Fig 3:**
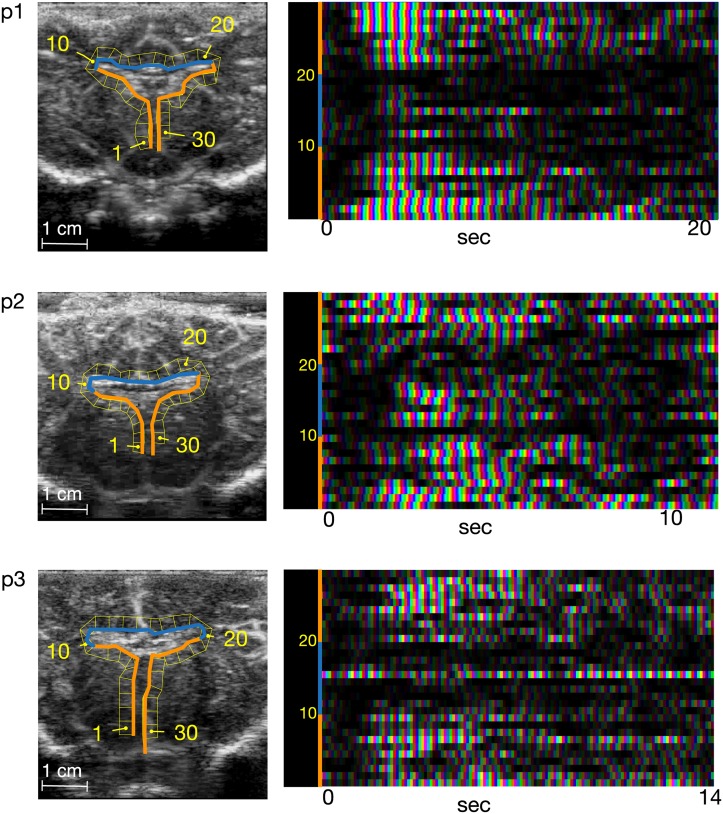
Peri-ventricular vascular pulse waves. The surface of the ventricle wall (first column) is unfolded to give the vertical axis of the running CF profile. The horizontal axis is time. Individual vascular PWs over the ventricular surfaces can be resolved.

The vascular PW distributions over the ventricle surfaces share rough temporal similarities among the three piglets (Fig 3). All three have a time profile consistent with the passage of an angiographic bolus. There is generally greater PW magnitude over the ventral surfaces despite a limited zone of high PW magnitude about the midline area of the ventral surfaces.

#### Arteriovenous ventricular surface PW relation

**Fig 4:**
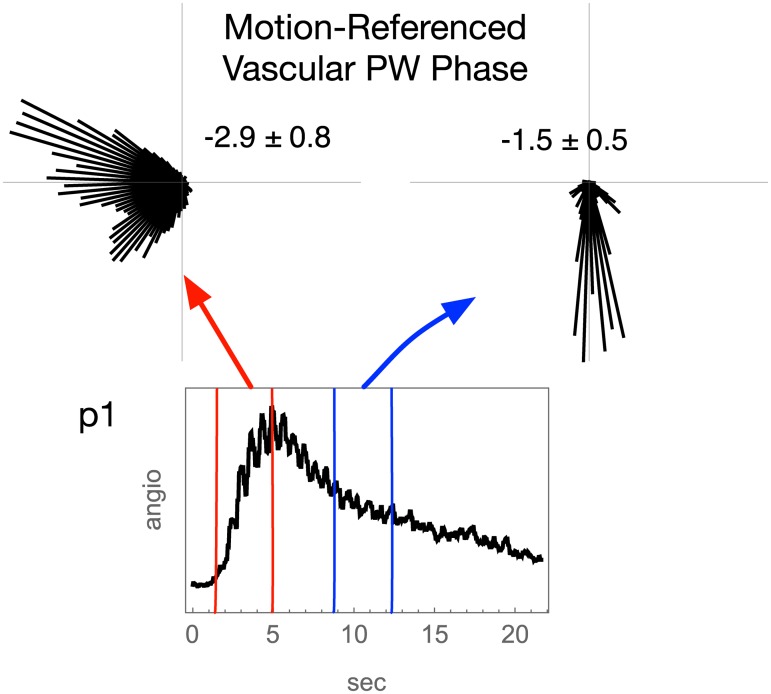
Peri-ventricular arterial and venous PW phase for p1. An angiographic time-intensity curve for the ventricular surface shows the inflow (arterial) and outflow (venous) segments of the bolus passage. Circular histograms pf vascular PW relative to brain pulse motion from arterial and venous segments of angiographic bolus travel (top row). The vascular PW phase data are from the wavelet vascular PW piglet images employing a time slice range of about 5 heartbeats as indicated by the red and blue line pairs in the angiographic time intensity curve (middle row). The angular mean and standard deviation are reported.

Circular histograms show that arterial and venous PW phase differ in relation to brain pulse motion, as shown for p1 in Fig 4 and for p2 and p3 in S4 Fig. The angular mean ± standard deviation are given for each in the figures. By Watson’s two-sample test for circular data, these distributions of phase angle difference appear not drawn from a common distribution, consistent with arteriovenous PW coupling (each have *p* ≤ 10^−5^ [6]).

#### Peri-ventricular PW distribution

**Fig 5:**
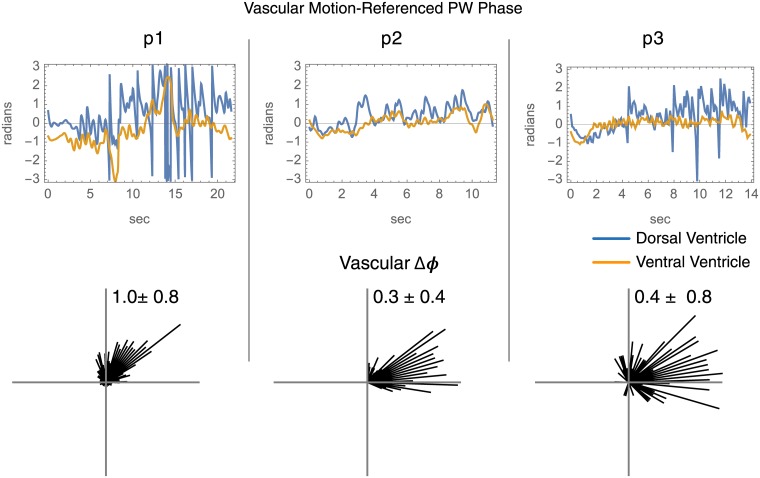
Peri-ventricular PW phase. The complex-valued vascular complex-valued PW data from the ventral (orange) and dorsal (turquoise) surfaces are referenced to pulse motion. In the upper row phase is plotted versus time for the dorsal and ventral peri-ventricular zones. The bottom row shows circular histograms of the phase differences between the two zones.

Fig 5 shows the vascular PW phase in relation to brain pulse motion in dorsal and ventral ventricular zones, and circular histograms of the aggregate dorsal versus ventral PW phase differences. For each piglet the mean phase difference differs from zero radians (*p* ≤ 10^−5^).

### Endoscopic third ventriculostomy in humans

#### Ventricle wall motion

**Fig 6:**
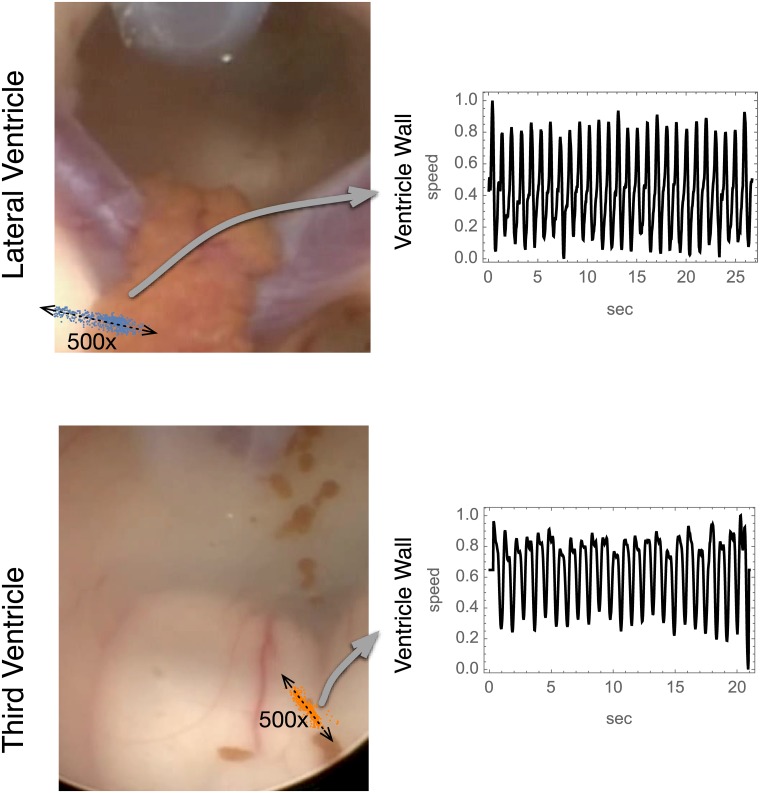
Ventricle wall velocity scattergrams superimposed On Neuro-Endoscopy images. This scattergram superposition illustrates the direction of ventricle wall motions in relation to anatomy. For clarity the wall excursions are lengthened by 500x. The one-dimensional wall speed is obtained from projecting the two-dimensional wall speed vectors onto the major axis of the scattergram indicated in each case by a dashed double arrow.

Fig 6 shows the ventricle wall motion scattergrams superimposed onto the corresponding neuro-endoscope images of the walls of the Lateral and Third Ventricles for h1. S1 and S2 Figs show the wall motion scattergrams for subjects h2 and h3. The variable orientation of the eccentricity of the scattergrams describes variability in the predominant direction of ventricle wall motion. These Figs also depict the generation of one-dimensional wall speed by projection wall velocity vector onto the major axis of the wall speed scattergram. Even after reduction to one-dimensional wall speed, the shape of the waveforms remains sufficiently varietal to impair a comparison of CF ventricle wall motion phenomena between dorsal and ventral ventricle compartments.

**Fig 7:**
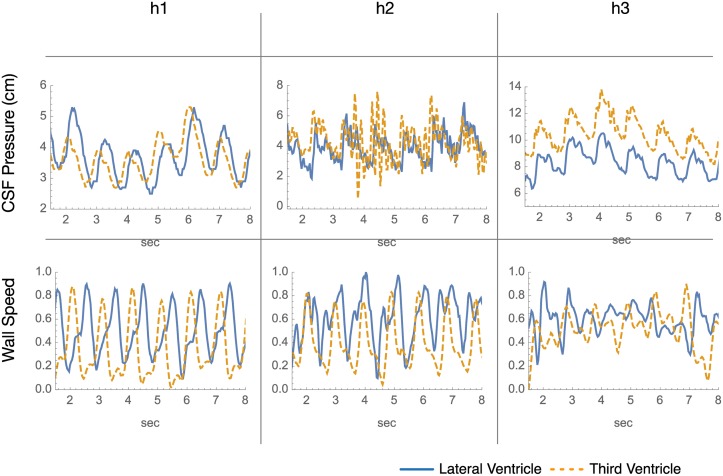
CSF pressure and wall speed in lateral and third ventricles. The waveforms in this Fig for the Lateral and Third Ventricles have been cardiac synchronized. To aid visual wave comparison the time span is reduced and baselines adjusted.

The CSF pressure waveforms have more similarity in shape between the Lateral and Third Ventricles than the wall speed signals (Fig 7). As a result, we focus on the comparison of CF phase differences in CSF pressure rather than wall speed.

#### Cardiac-synchronized lateral and third ventricle wall speed and CSF pressure waveforms

When the ventricle wall speed and CSF pressure signals in the Lateral and Third Ventricles are synchronized by reference to the peripheral blood pressure signal or to ECG, a slight but consistent phase difference is seen in the circular histograms of phase difference, with the Third Ventricle peaks tending to occur slightly before the Lateral Ventricle peaks (Fig 7).

#### Phase difference in wavelet-filtered cardiac synchronized CSF pressure waveforms

**Fig 8:**
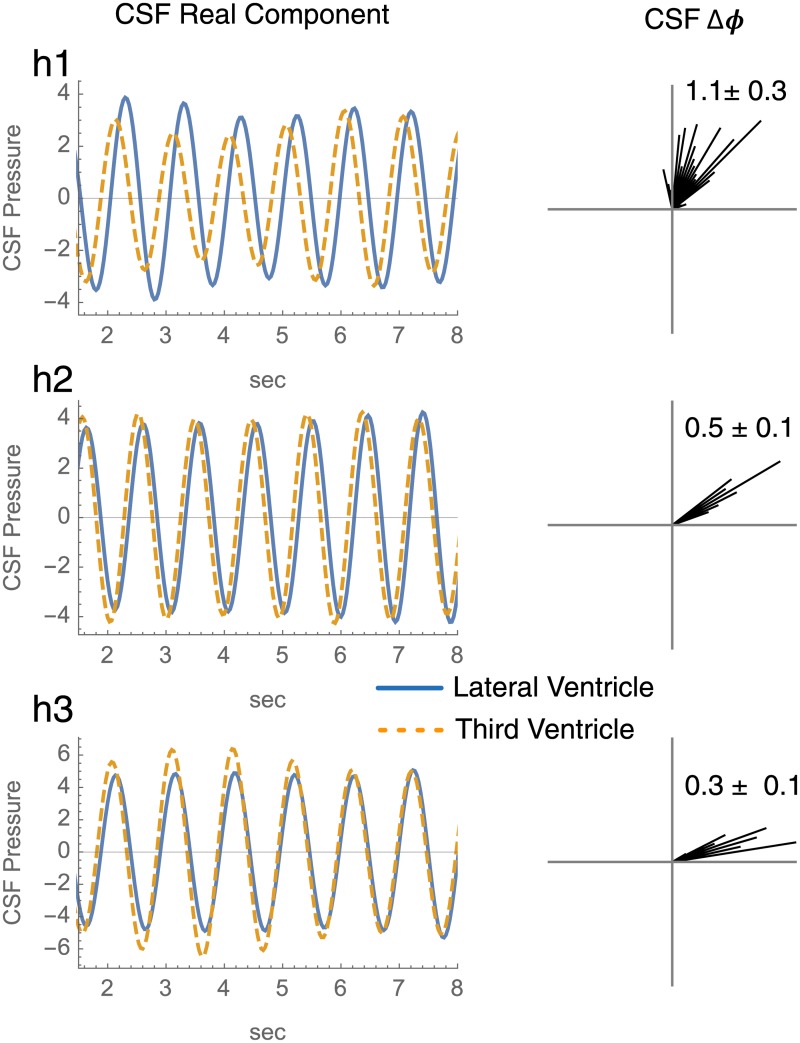
Phase differences of CSF pressure waveforms between lateral and third ventricles in humans after cardiac synchronization. The left column gives a limited duration of the real components of the Gabor wavelet filtered Lateral (turquoise) and Third Ventricle (orange) CSF pressure waveforms. The right gives the a histogram for the phase differences in each. The angular values are given in radians as mean ± standard deviation.

The Lateral and Third Ventricle CSF pressure waveforms are wavelet filtered for CF phenomena, and their phase differences after cardiac-synchronizationare computed by a cross-correlated wavelet technique (Fig 8) [9–12]. The computed phase differences are displayed as circular histograms. For each subject, the distribution of phase differences has a mean that differs from 0 radians (Watson’s large sample nonparametric test *p* ≤ 10^−5^ [7]).

### Morphologically matched dorsal-ventral phase differences

**Fig 9:**
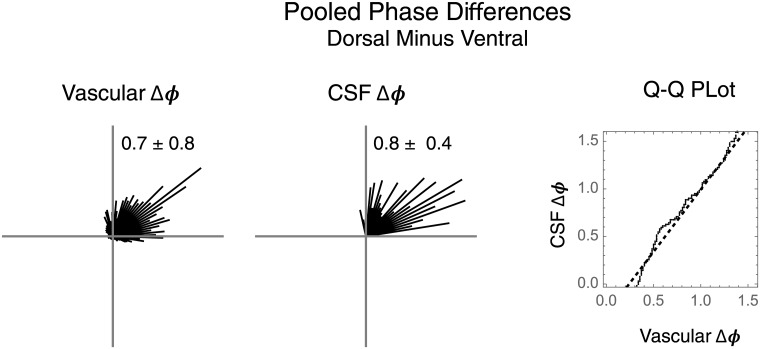
Dorsal-ventral pooled phase differences. The piglet vascular PW phase differences are pooled from Fig 5. The human CSF phase differences are pooled from Fig 8. The angular mean and standard deviation are reported. The vascular and CSF phases distributions are matched by quantile to give the Q-Q plot.

The vascular and CSF phase differences may be pooled each into a circular histogram (Fig 9) for side by side inspection. When the PW data about the dorsal and ventral surfaces of the ventricles are pooled, the phase of the difference of means is 0.7 ± 0.8 radians (Fig 9). This phase difference is consistent with the arrival of a vascular PW to the ventral ventricular zone prior to the dorsal zone. The the phase differences in CF CSF pressure variation are pooled, the phase of the difference of the means is 0.8 ± .4 radians. The signs of the phase differences imply the presence of vascular and CSF PWs that travel from ventral to dorsal in direction. The pooled phase histograms may be joined by quantiles into a single Q-Q plot (Fig 9) [14]. The similarity of the dorsal minus ventral vascular and CSF phase difference distributions in the Q-Q plot does not allow us to reject the possibility of a spatial co-relationship between them.

## Discussion

For the three piglets, the vascular PW phase over the ventricle surfaces is different in the arterial versus venous segments of the angiographic time intensity curve (Fig 4 and S4 Fig). This is consistent with arteriovenous PW coupling in the peri-ventricular zones. For the three piglets, the peri-ventricular vascular PWs share a consistent dorsal versus ventral phase gradient (Fig 9). This implies a consistent spatial distribution of vascular PW phase over the surfaces of the ventricles wherein vascular PWs travel from ventral to dorsal.

For the three humans, the intra-ventricular CSF pressure variations at CF share a consistent spatial phase gradient between the dorsally located Lateral Ventricle and the ventrally located Third Ventricle (Fig 9). This is consistent with the presence of a consistent spatial distribution of CF variations of CSF pressure within the ventricles, wherein CSF travel from ventral to dorsal.

When morphologically matched (Fig 9), the human endo-ventricular and piglet peri-ventricular data are spatially consistent with the interpretation that vascular PWs deform the ventricles and induce adjacent CSF pressure variations. The acquisition of the vascular and CSF data in different subjects does not permit an argument of causality. Nonetheless, these exploratory data fail to falsify the hypothesis that CSF as configured by the ventricles behaves as a relay medium for arteriovenous PW coupling.

### Ventricular CSF as pulse wave relay medium

What advantages might CSF offer over brain tissue as a low viscosity PW relay medium? A lower viscosity fluid flows faster and takes more quickly the shape of a container than a high viscosity fluid. This should

1. allow the change in shape of a brain ventricle wall produced by an arterial PW to generate a local CSF motion more quickly;
2. allow a remote ventricle wall to change shape more quickly in response to an adjacent CSF motion; and
3. permit lower friction CSF mass transfer between these reciprocally moving ventricle wall sites.

The net effect of CSF should be to extend the spatiotemporal reach of PW coupled information exchange and to reduce the friction in the adaptation to irregular CF brain shape changes. This might for example allow the brain better to accommodate local heterogeneities of arterial and venous blood volume in the overall match of an arriving arterial stroke volume with venous blood ejection. Further study might include the development of experimental models where ventricular CSF is substituted for liquids of varying viscosity.

If a PW conducted via CSF were to travel sufficiently fast compared to an arterial PW it could enable PW coupling between the local peak and remote trough of an arterial PW. This putative arterio-arterial PW coupling, which is not experimentally examined in this paper, requires that PW coupling be remote whereas arteriovenous PW coupling remains effective when local.

### Limitations

The main limitation of reliance on a morphological and a cardiac cycle match of the ventricles between piglets and humans awaits the introduction of methods for the simultaneous observation of vascular PW travel, ventricle wall motion, and adjacent CSF pressure variations. We concede that the morphological match is necessarily only approximate, as these belong to different mammalian orders. In the human the Lateral Ventricle appears proportionately larger compared to the piglet. The ventral surface of the Lateral Ventricle in the piglet as defined in this paper extends across the foramen of Monro to form part of the wall of the Lateral Ventricle. Nonetheless, the human and the piglet ventral-dorsal ventricle zones evaluated here share a directionally consistent ventral-dorsal direction of phase gradient.

This hypothesis assumes that a vascular PW gradient generates a force gradient in the ventricle walls that drives a CSF pressure gradient but we have not measured the biophysical properties of the structures that convey the forces between the vasculature and the ventricle walls, nor have we measured the fluid dynamics between the ventricle wall motions and the nearby CSF variations. We have not measured the propagation velocity of the vascular PW over the surface of the ventricles in piglets nor the motion velocity of the contained CSF in humans. We make the assumption that ventricle wall motion is mainly influenced by local peri-ventricular vascular PWs but our choice of the thickness of the peri-ventricular region of interest is arbitrary. When we commenced the experiments we had intended to analyze fully the direction and phase of ventricle wall motions, but find their complexity to be intractable to methods available to us.

Our hypothesis of arteriovenous PW phase complementarity does not have the refinement to predict a numerical phase difference range, only that by conservation of mass the high volume phases of arterial and venous PWs should not coincide. The timing relationship between peri-ventricular peak arterial and peak venous PW differs among the three piglets (Fig 4 and S4 Fig), possibly reflecting different mixtures of local versus remotely relayed PW coupling. The related article reports an arteriovenous PW phase difference over the entire imaged piglet coronal view [2]. We cannot exclude based on these data the possibility that the observed ventricular surface arteriovenous phase difference has no special local meaning but is only a reflection of a larger and more systematic arteriovenous phase difference across the entire brain.

The ultrasound angiography technique employed here yields 2-D images in planar geometry. Evolving methods ultrasound imaging methods with increased sensitivity, sampling speed, and 3-D capability might extend the results reported here [15, 16]. In particular, if vascular PWs were to be imaged in 3-D, we would predict that vascular PWs would propagate with consistent timing and spatial distribution over the entire imaged ventricular surface.

### Ependymal cilia

The visual observation reported here of CF ventricle wall motions and CSF pressure variations point to the heart rather than to ependymal cilia as the driver of intraventricular CSF flow [17]. The CF CSF motions produced ultimately by the heart beat as observed here may instead produce physiologically meaningful deflections of ependymal cilia. [18–20].

Several workers have reported a notch filter phenomenon in intracranial pressure (ICP) measurements whereby an ICP trace appears to have unexpectedly attenuated CF signal compared to other frequencies and attribute to the ventricles a pulsation absorber characteristic [21–23]. The findings reported here do not support the interpretation of the ventricles as terminal pulsation absorbers but suggest instead how they could lead to a similar experimental result by serving as a relay medium for arteriovenous PW coupling.

### Hydrocephalus and other disturbances of intracranial pressure

These data suggest that the ventricles play a role in arteriovenous coordination by configuring CSF to act as a low viscosity relay medium for vascular PW coupling. The absence of an understanding of the purpose of and mechanism of action behind the ventricles otherwise continues to impair progress in the management of disturbances of intracranial pressure including hydrocephalus, a clinical disorder of the ventricles of the brain that affects about 1 in 1,000 live births but can develop at any age, and can be disabling and even fatal [24].

## Supporting information

S1 Fig Lateral and Third Ventricle neuro-endoscope views with CSF and wall speed data for h2. (PDF)

S2 Fig Lateral and Third Ventricle neuro-endoscope views with CSF and wall speed data for h3. (PDF)

S3 Fig Video and CSF pressure synchronization. Snapshots of the graphical user interface of the alignment program one video frame apart (left) just before and (right) after contact of operator’s finger with the catheter. Finger contact with the catheter defines a common reference time point in the CSF pressure waveform and the endoscope video.
(PDF)

S4 Fig Peri-ventricular arterial versus venous PW phase for p2 and p3. (PDF)

S1 Video Example video of GUI program for synchronizing video with physiology by video capture of a finger flick of a pressure-transduced catheter. (MP4)

S2 Video Raw video neuro-neuroendoscopy data for h1. (MOV)

S3 Video Raw Video Neuro-Endoscopy data for h2. (MOV)

S4 Video Raw Video Neuro-Endoscopy data for h3. (MOV)

S5 Video Raw ultrasound angiography data for p1 per S1 File. (MOV)

S6 Video Wavelet PW reconstruction for p1 per S1 File. (MOV)

S7 Video Raw ultrasound angiography data for p2 per S1 File. (MOV)

S8 Video Wavelet PW reconstruction for p2 per S1 File. (MOV)

S9 Video Raw ultrasound angiography data for p3 per S1 File. (MOV)

S10 Video Wavelet PW reconstruction for p3 per S1 File. (MOV)

S1 Data Raw Physiology Data for h1. (CSV)

S2 Data Raw Physiology Data for h2. (CSV)

S3 Data Raw Physiology Data for h3. (CSV)

S1 File Wavelet brain angiography suggests arteriovenous pulse wave phase locking (companion manuscript). Those raw and wavelet-reconstructed angiographic data in the companion manuscript that are subjected to further analysis in this paper are provided in the next six video files.
(PDF)
