# Supplementary figures and images for: CSF in the ventricles of the brain behaves as a relay medium for arteriovenous pulse wave phase coupling
Source: PLoS One. 2017 Nov 15;12(11):e0181025. doi: 10.1371/journal.pone.0181025 (PMC5687699; doi:10.1371/journal.pone.0181025)

Lateral Ventricle

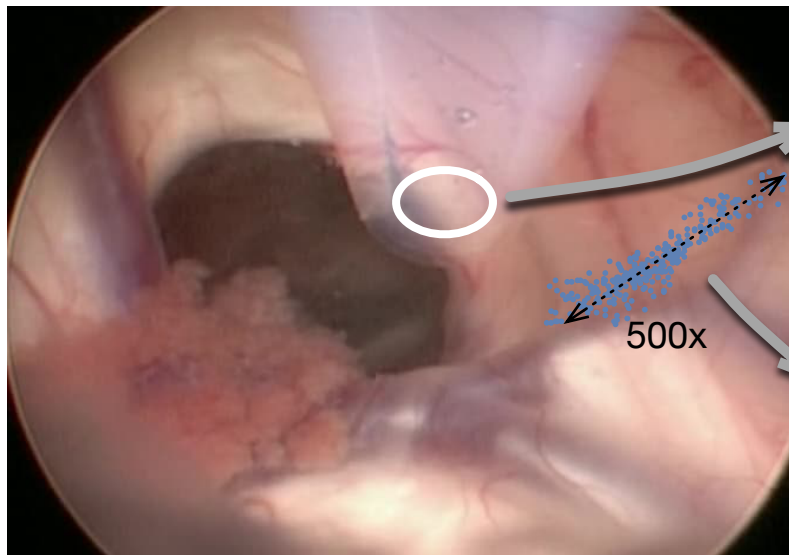

CSF Pressure

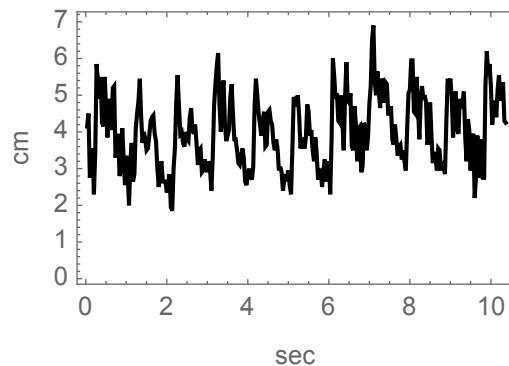

Ventricle Wall

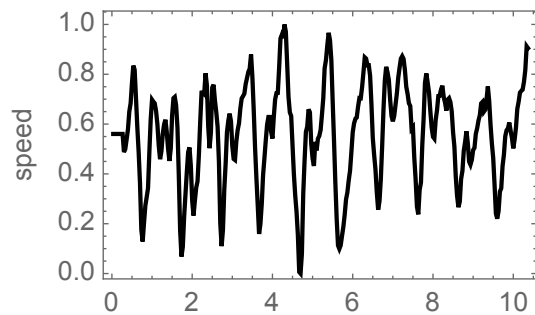

Third Ventricle

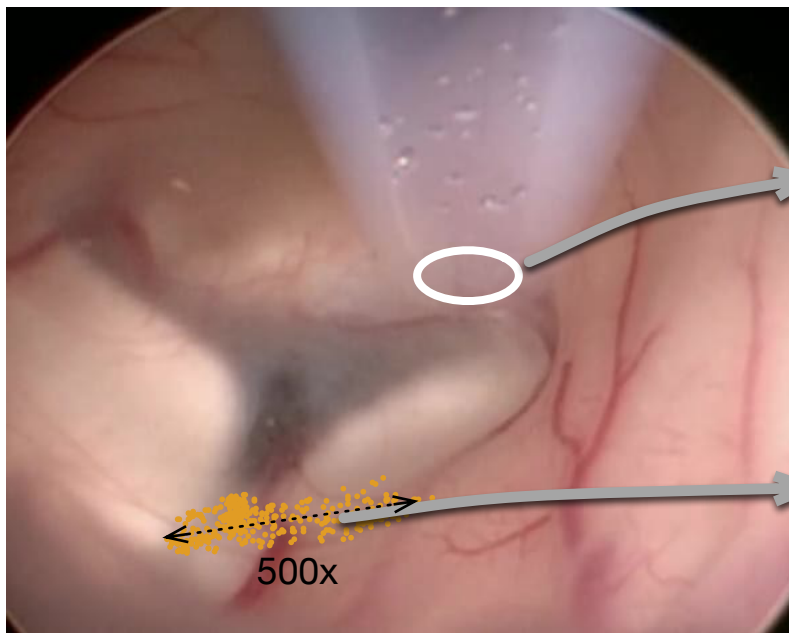

CSF Pressure

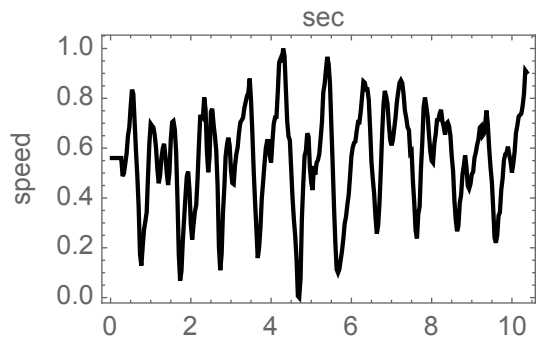

Ventricle Wall

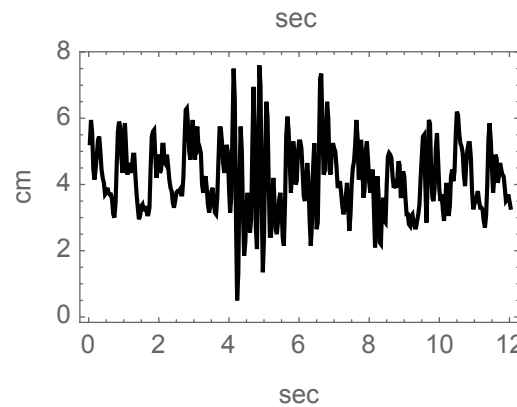

Supplement: S1 Fig — (PDF) [file pone.0181025.s001.pdf]

Lateral Ventricle

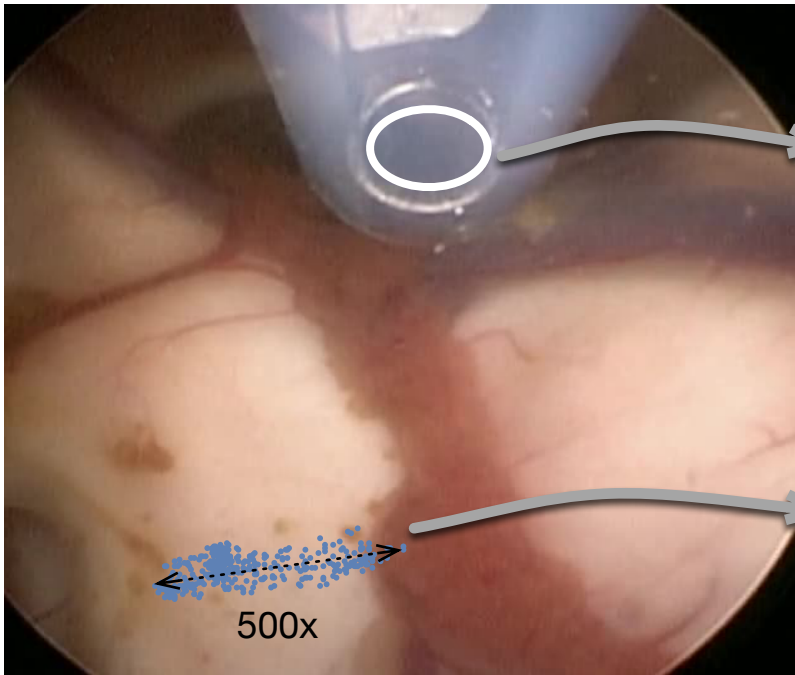

CSF Pressure

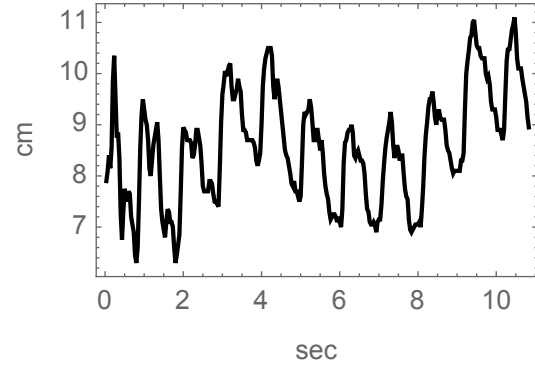

Ventricle Wall

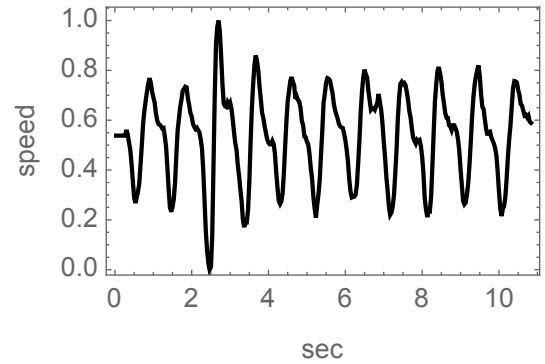

Third Ventricle

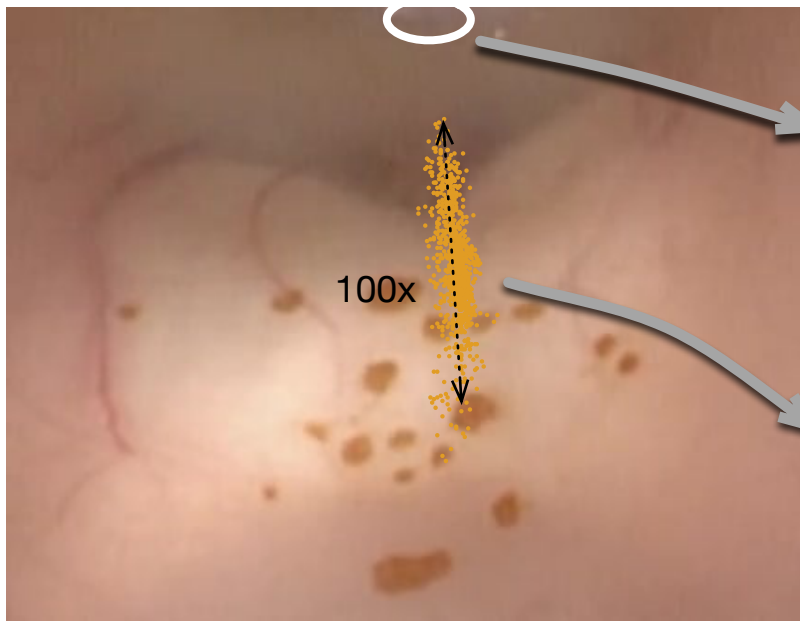

CSF Pressure

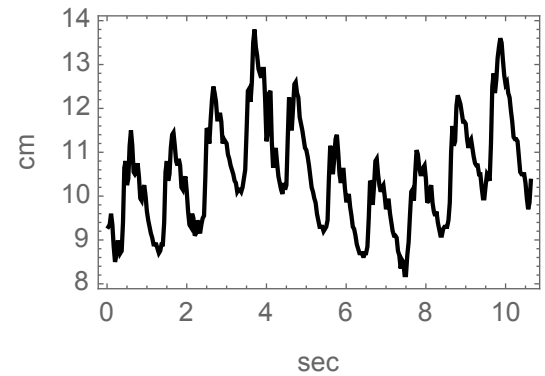

Ventricle Wall

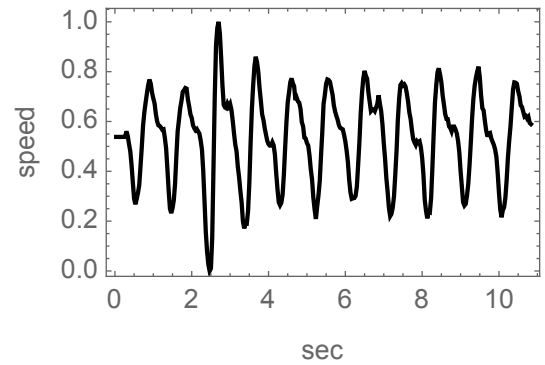

Supplement: S2 Fig — (PDF) [file pone.0181025.s002.pdf]
