# Supplementary material for: CSF in the ventricles of the brain behaves as a relay medium for arteriovenous pulse wave phase coupling
Source: PLoS One. 2017 Nov 15;12(11):e0181025. doi: 10.1371/journal.pone.0181025 (PMC5687699; doi:10.1371/journal.pone.0181025)
Supplement: S3 Fig — Snapshots of the graphical user interface of the alignment program one video frame apart (left) just before and (right) after contact of operator’s finger with the catheter. Finger contact with the catheter defines a common reference time point in the CSF pressure waveform and the endoscope video. (PDF) [file pone.0181025.s003.pdf]

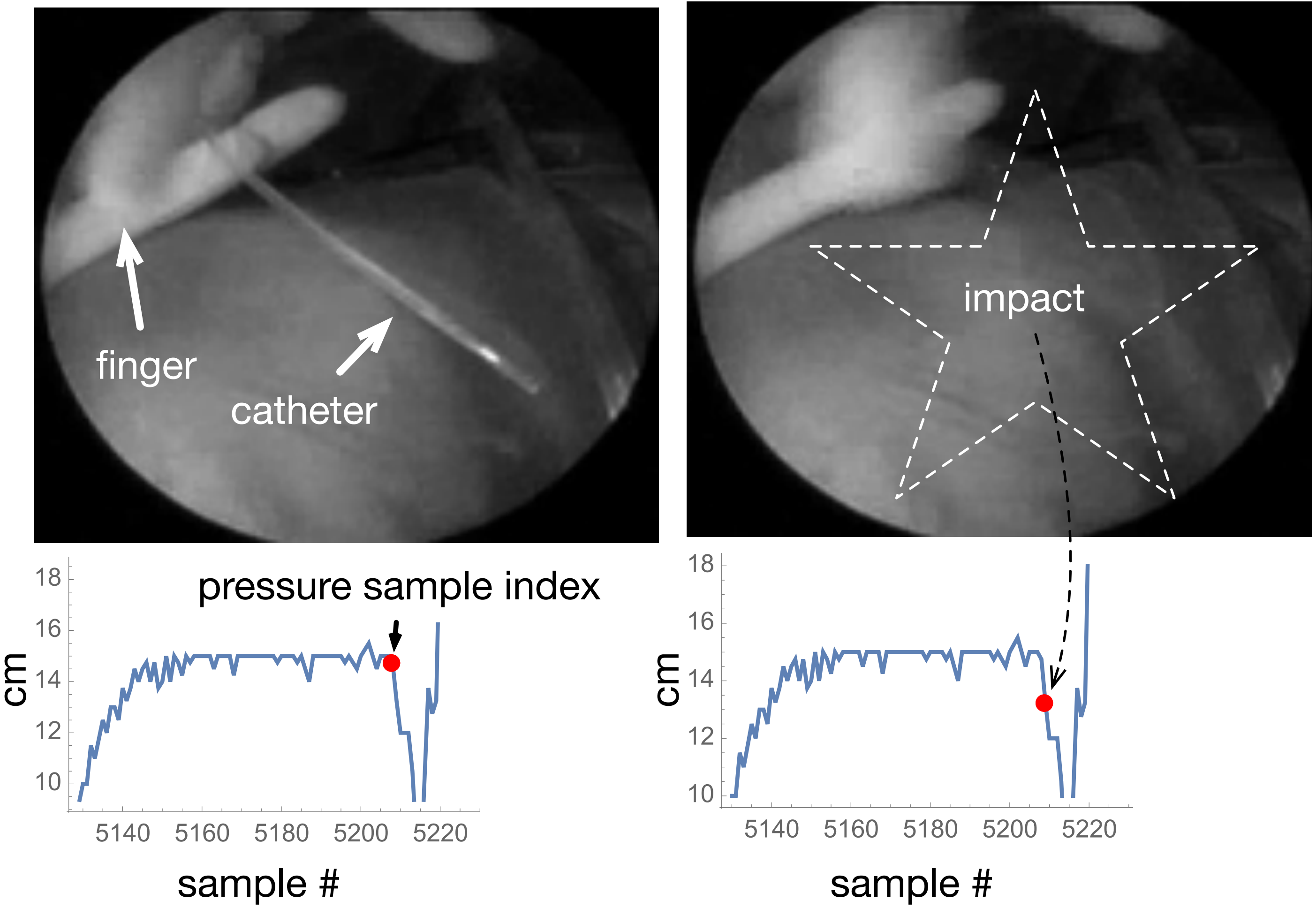

Video and CSF Pressure Synchronization. Snapshots of the graphical user interface of the alignment program one video frame apart (left) just before and (right) after contact of operator's finger with the catheter. Finger contact with the catheter defines a common reference time point in the CSF pressure waveform and the endoscope video.
