# Supplementary material for: CSF in the ventricles of the brain behaves as a relay medium for arteriovenous pulse wave phase coupling
Source: PLoS One. 2017 Nov 15;12(11):e0181025. doi: 10.1371/journal.pone.0181025 (PMC5687699; doi:10.1371/journal.pone.0181025)
Supplement: S4 Fig — (PDF) [file pone.0181025.s004.pdf]

Motion-Referenced Vascular PW Phase

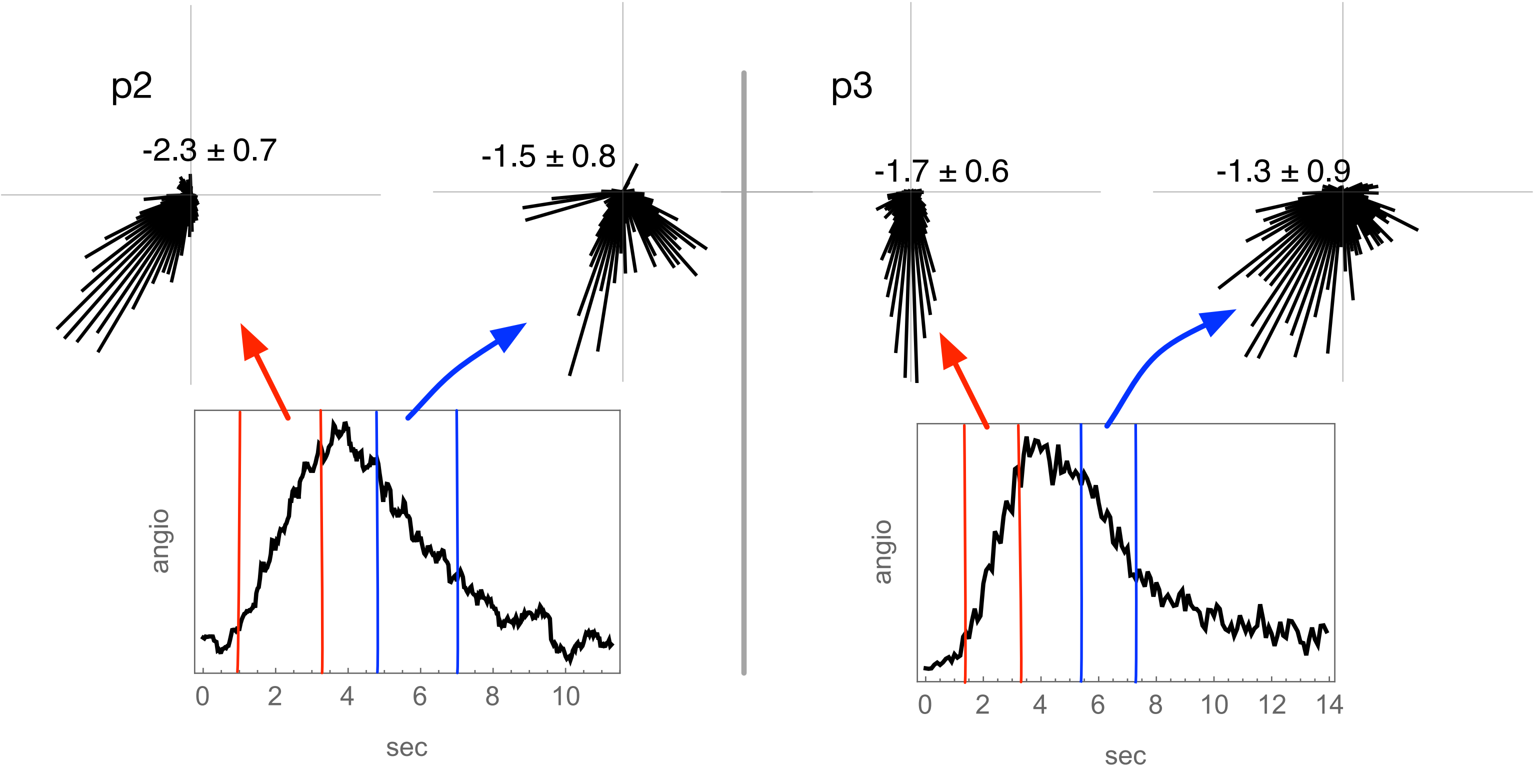

Peri-ventricular arterial versus venous PW phase for p2 and p3.
