# Supplementary material for: CSF in the ventricles of the brain behaves as a relay medium for arteriovenous pulse wave phase coupling
Source: PLoS One. 2017 Nov 15;12(11):e0181025. doi: 10.1371/journal.pone.0181025 (PMC5687699; doi:10.1371/journal.pone.0181025)
Supplement: S1 File — Those raw and wavelet-reconstructed angiographic data in the companion manuscript that are subjected to further analysis in this paper are provided in the next six video files. (PDF) [file pone.0181025.s018.pdf]

Wavelet Brain Angiography Suggests Arteriovenous Pulse Wave Phase Locking

Willian E. Butler[1,*]

1 Massachusetts General Hospital, Neurosurgical Service, Boston, Massachusetts 02114, USA

* wbutler@mgh.harvard.edu

Abstract

When a stroke volume of arterial blood arrives to the brain, the total blood volume in the bony cranium must remain constant as the proportions of arterial and venous blood vary, and by the end of the cardiac cycle an equivalent volume of venous blood must have been ejected. I hypothesize the brain to support this process by an extraluminally mediated exchange of information between its arterial and venous circulations. To test this I introduce wavelet angiography methods to resolve single moving vascular pulse waves (PWs) in the brain while simultaneously measuring brain pulse motion. The wavelet methods require angiographic data acquired at significantly faster rate than cardiac frequency. I obtained these data in humans from brain surface optical angiograms at craniotomy and in piglets from ultrasound angiograms via cranial window. I exploit angiographic time of flight to resolve arterial from venous circulation. Initial wavelet reconstruction proved unsatisfactory because of angiographic motion alias from brain pulse motion. Testing with numerically simulated cerebral angiograms enabled the development of a vascular PW cine imaging method based on cross-correlated wavelets of mixed high frequency and high temporal resolution respectively to attenuate frequency and motion alias. Applied to the human and piglet data, the method resolves individual arterial and venous PWs and finds them to be phase locked each with separate phase relations to brain pulse motion. This is consistent with arterial and venous PW coordination mediated by pulse motion and points to a testable hypothesis of a function of cerebrospinal fluid in the ventricles of the brain.

Introduction

The forces behind brain pulsations arise in the heart, which pumps blood to the brain in a sequence of stroke volumes. When an arterial stroke volume arrives to the intracranial compartment, a hemodynamic variation spreads through the cerebrovascular tree and induces brain motion. There are constraints to this process, and I hypothesize that these constraints oblige the brain to handle an arriving stroke volume with an orderly pattern of brain pulse motion, arterial pulse waves (PWs), and venous PWs. To test this hypothesis, I develop wavelet imaging methods to time resolve the joint cardiac frequency (CF) processes of brain motion and hemodynamic variation.

An example constraint is that an arriving arterial stroke volume ought to displace from within the bony cranium an approximately equal volume of venous blood across the cardiac cycle [1]. I hypothesize that to coordinate this displacement, an arriving stroke volume of arterial blood somehow induces a transfer of information across

extravascular spaces to the venous circulation. The transfer of information may be mediated by solid brain tissue, by cerebrospinal fluid (CSF) including in the ventricles of the brain, or cooperatively by both. I hypothesize that the net transfer of information from the arterial side to the venous side enables the measured ejection from the cranium of an appropriate volume of venous blood coupled with an appropriate quantity of kinetic vascular energy. This way in a given cardiac cycle there is a match of the volumes and kinetic energies after frictional losses of blood moving in and out of the cranium. It is unlikely that there is sufficient CF information exchanged through the capillaries to coordinate this balance because experimental cranial window microscopy studies have not found pulsatile velocity changes of red blood cells in brain capillaries [2].

This paper focuses then on the topic of tissue pulse motion and its interaction with arterial and venous PWs within that tissue. A companion manuscript explores a potential contribution to the information transfer by CSF in the ventricles of the brain (Supporting Information File SI-55).

Those CF phenomena that show coherence, meaning relative spatial uniformity of CF phase, are defined in this paper to represent a PW. The modifier "pulse" signifies CF timing.

Whether an arriving arterial stroke volume ejects a matching volume of venous blood by simple conservation of mass, meaning for example that an arterial wall diameter expansion mechanically causes venous wall contraction, or whether active regulatory systems participate is beyond the scope of this paper. In either case, the hypothesis that there is coordination among tissue pulse motion and arterial and venous PWs experimentally predicts that

- Arterial blood in the brain forms a PW.
- Venous blood in the brain forms a PW.
- There is CF brain motion.
- These separately resolvable CF phenomena maintain coordinated phase relations.

Wavelets are mathematical objects that may enable the time-resolved extraction of CF phenomena. If the sampling rate is fast enough, wavelet methods should allow the deconstruction of events at a fine temporal resolution within each given of a sequence of cardiac cycles. Wavelet methods require that the underlying non-sparse angiographic data be acquired at a sampling rate significantly higher than CF, as governed by the sampling theorem of Nyqvist, Shannon, and Kotelnikov [3–6]. I report here data from human brain surface optical angiography and from piglet cranial window ultrasound angiography. Both of these angiographic methods allow at 30 Hz the acquisition of angiographic images of a passing bolus of contrast simultaneous with the observation of brain motion.

To classify a vascular PW as arterial or venous, I exploit the time of flight of a sharply delivered angiographic bolus, and assume vascular PWs that occur early to be arterial and those that occur late to be venous.

Cardiac Gated Imaging Methods

Under certain circumstances, CF phenomena in the brain may be imaged by methods such as such as computed tomography (CT) and magnetic resonance imaging (MRI) even though those methods sample at slower than the cardiac rate by the use of cardiac gating [7–10]. In that approach, the timing of individual images is matched to an electrocardiogram (ECG) signal to enable the eventual interpolation of images within

an averaged cardiac cycle [11, pp 443-454]. Unlike the methods reported here, cardiac gated imaging methods do not image directly a chain of events internal to a given cardiac cycle. Instead, cardiac gating relies on the assumption that all heart beats are alike. Similarly, gated image sequences cannot capture variant phenomena that span from one heart beat to the next. Experimental methods that focus on physiological signals such as blood pressure and intracranial pressure (ICP) have the temporal resolution to register single CF events but since they do not offer images they are limited in their ability to portray the spatial basis of causal relations [12–14].

Materials And Methods

The human brain surface optical and piglet cranial window ultrasound angiography methods employed here share the properties of (1) capturing angiographic images at a rate significantly greater than CF, (2) permitting the concurrent measurement of brain motion, and (3) offering angiographic time of flight (ATOF) to separate arterial from venous circulation.

Human Brain Surface Optical Angiography

Subjects

I inspected archived optical angiograms of 11 personally treated aneurysm craniotomy undergoing craniotomy for a cerebral aneurysm to reduce the risk of aneurysmal re-hemorrhage. These human patients were managed according to clinical conventions [15]. No aspect of their care was altered for the purpose of this study. There is no attempt in this study to comment on the clinical value of intraoperative human brain surface optical angiography, nor is there any intent to suggest whether or not the methods developed here have any potential for clinical utility. The human portion of this study is limited to the post-hoc analysis of archived data routinely collected in the course of clinical care. The data have no individually identifying features. Hence, the institutional committee for research involving human subjects waived the requirement to approach the subjects for consent.

There are steps commonly taken in such intraoperative optical angiograms to optimize their clinical decision making value that happen to undercut their scientific value for wavelet analysis. For example, since optical angiography is a line of sight technique, to fully visualize all of the vasculature in the vicinity of an aneurysm, in most of my operated cases I move the microscope actively or manually manipulate tissues during the angiogram in order to see behind them. In addition, since the vasculature of interest is arterial, optical angiographic recording may be halted before entry into the venous phase. Of those, optical angiograms in four human subjects (designated h1-4) met criteria for wavelet analysis. Their mean age at the time of surgery was 64.5 years (range 59 to 76 years). There was no optical angiogram were wavelet analysis was commenced but completed.

Procedure

At craniotomy under general anesthesia, the aneurysm and associated vessels are exposed with microsurgical technique with an operating microscope that is equipped to perform intraoperative indocyanine green angiography (Carl Zeiss Surgical Microscope OPMI Pentero INFRARED 800, Carl Zeiss Meditec AG, Jena, Germany). Generally these aneurysms lay in clefts between the lobes and fissures of the brain, and the surrounding brain surface is neocortex formatted as gyri and sulci (Supporting Information File SI-1 Fig XF1). A temporary clip is applied to the aneurysm's major

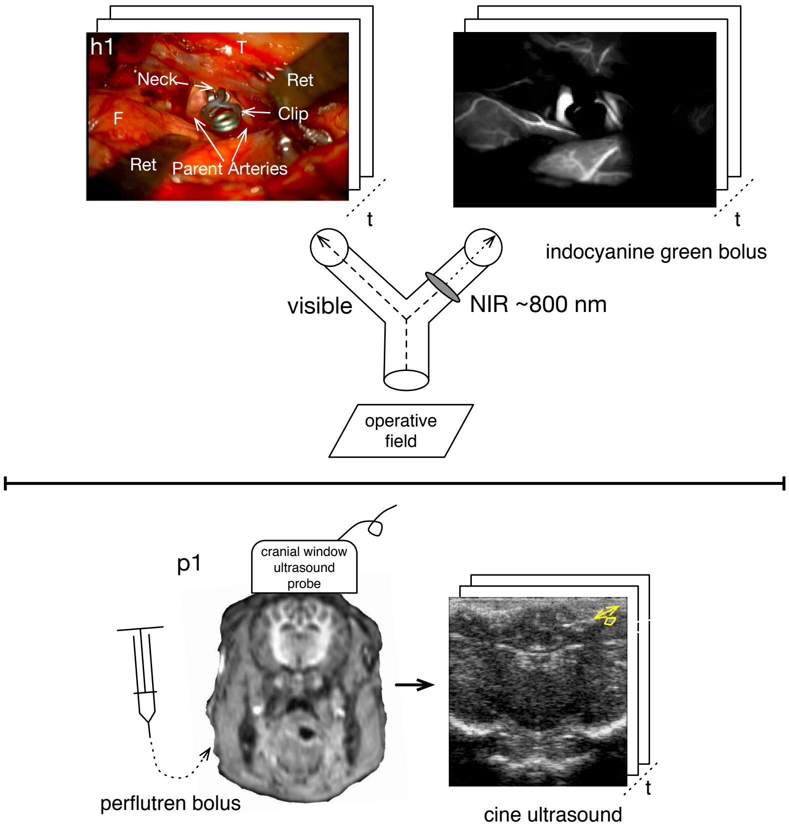

Fig 1. Angiography Sampled Faster Than Cardiac Rate. Upper panel is human brain surface optical angiography with simultaneous visible recording via a beam splitter. The aneurysm is of the right middle cerebral artery, where F=frontal lobe, T=temporal lobe, and Ret=retractors. The lower panel is piglet ultrasound angiography via cranial window.

feeding artery, the neck of the aneurysm undergoes final dissection, a clip is applied across the neck to exclude the aneurysm from blood flow while not including apart of the parent artery in the clip since that would impair blood flow through it. The temporary clip on the major feeding artery is removed.

At this point the intraoperative optical angiogram is performed to confirm that the clipped aneurysm no longer fills with blood and that the associated vessles have not been inadvertently occluded by the clipping [16–20]. On signal, the anesthesiologist injects intravenously 25 mg of indocyanine green (ICG) and the surgeon presses a sterilely covered control button on the operating microscope to trigger the video recording through the microscope of two optical channels via a beam splitter. One channel records visible wavelength, and other contains a near infrared (NIR) pass filter that records the ICG fluorescence and thereby yields NIR ICG video-angiogram (Fig 1). The two video recordings, captured each at 30 Hz, are prepared for analysis as described in Supporting Information File SI-1 Section 1.2.

Piglet Cranial Window Ultrasound Angiography

While ultrasound can image at a faster than cardiac rate, the blockage of sound by the skull bone impedes its sensitivity for this purpose [21]. In the piglet model reported here, the angiographic ultrasound imaging is through a cranial window of a passing

bolus of perflutren, an ultrasound contrast agent [22]. The same ultrasound cine images allow the measurement of tissue motion by the tracking of translations of discrete brain structures (Fig 1).

Subjects

Ultrasound angiography though a cranial window was performed in three piglets weighing 10 kg (designated p1-3) with institutional animal subjects committee approval. In one of them, the cranial window experiment was performed in an MRI suite, offering near simultaneous high anatomic resolution.

Procedure

Prior to the piglet experiments, the signal characteristics of the contrast agent, perflutren, was tested in dynamic phantoms and found to be suitable for these purposes. This is further described in Supporting Information File SI-1 Section 2.1.

Under the care of a veterinarian, the piglets were premedicated with intramuscular xylozine 2 mg/kg then general anesthesia was induced with an intramuscular tiletamine/zolazepam combination and maintained with inhalational isoflurane. ECG leads, a peripheral pulse oximetry monitor, and a femoral vein line were applied to each of the three anesthetized piglets with the animals positioned supine. Each animal was then turned prone, and with the head rigidly held above the level of the heart a craniectomy centered on the coronal suture was performed of the minimal size required to allow direct application of the ultrasound probe to the dura. The ultrasound probe was held firmly in place during imaging by a rigid mechanical arm (lower panel of Fig 1).

After acquiring baseline cine images, an activated vial containing 1.2 million microspheres was manually injected sharply into the femoral vein catheter and cine images of the intracranial cavity were recorded until the bolus of contrast had passed and steady state achieved (approximately 20 seconds). In one experiment, the animal was transported immediately after ultrasound imaging to a 3-Tesla MRI unit. The magnetic resonance images serve to confirm anatomic orientation of the ultrasound images. The post-gadolinium images do not show abnormal contrast enhancement. The animals were maintained under general anesthesia throughout, then euthanized while still under anesthesia with an intravenous pentobarbital overdose.

The image spatial dimensions and sampling characteristics are presented in Supporting Information File SI-1 Table XT2.

Pulse Motion Measurement

Both of the imaging methods of this paper, optical imaging of human brain surface and cranial window ultrasound in a piglet, provide an opportunity for concurrent imaging of angiographic phenomena and brain motion. With the human brain surface optical data, the brain motion is measured on the visible wavelength recording whereas in the piglet cranial window ultrasound it is measured on the same same recording that shows the angiographic contrast passage. The two methods are otherwise identical and pulse brain motion is computed for the human and piglet data with the same source code. A region of interest (ROI) for motion tracking was drawn on an image of a video sequence, and the 2D translations of the ROI that maximize their correlation were taken to represent the motion.

The regions selected for pulse motion tracking in human brain surface optical angiography are shown for h1 in Fig 2 and for h2-4 in Supporting Information File SI-1 Fig XF4. Freeze frame images of the ultrasound video sequences along with the regions

selected for pulse motion tracking are shown for p1 in Fig 2 and for p2-3 in Supporting Information File SI-1 Fig XF7.

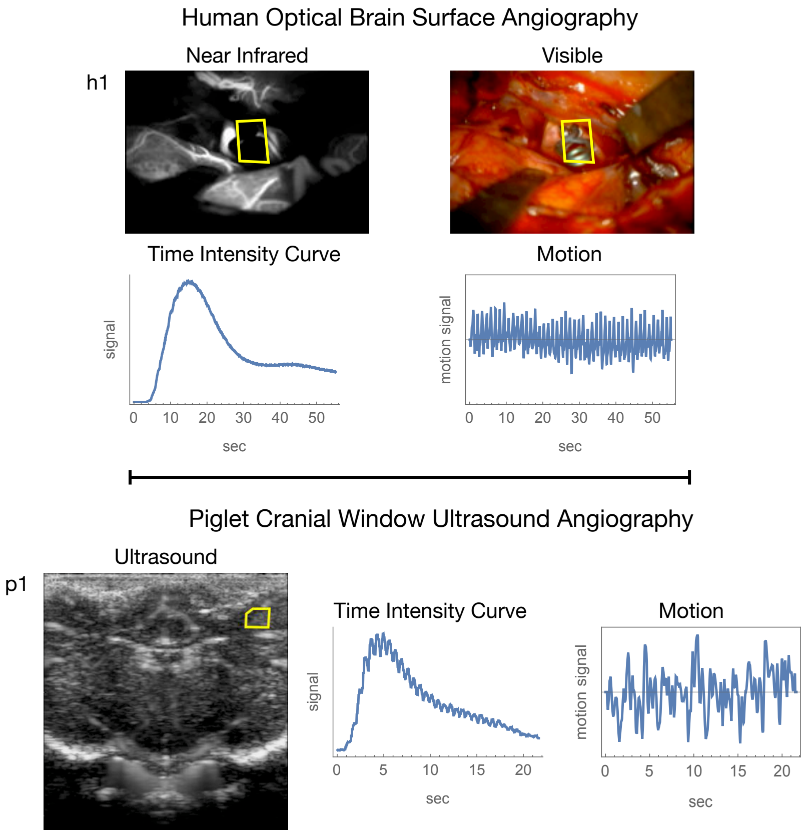

Fig 2. Representative Snapshot Images And Temporal Data Summaries For Human Subject h1 And Piglet Subject p1. The yellow polygon in the images gives the region for motion tracking.

Wavelet Angiography

Time Frequency Resolution

Complex-valued wavelets have properties useful for mapping the temporal and spatial behavior of CF angiographic phenomena [23–25]. Unlike an unmodified Fourier transform, a wavelet transform can represent time varying CF phenomena. In addition, a complex number may be rendered conveniently as a pixel in a raster computer display by using polar coordinates in a brightness-hue color model. If $c = a + i\,b$ represents a complex-valued CF datum in a spatiotemporal grid with real component a and imaginary component b, it has a polar representation $\{|c|, \phi_c\}$ where magnitude $|c| = \sqrt{a^2 + b^2}$ may be rendered in a pixel as brightness and ϕ_c, the angle between the positive x-axis and the point $\{a, b\}$, as hue (Supporting Information File SI-1 Fig XF9).

Whereas Fourier methods make reference to a signal's frequency, by convention wavelet methods make reference to scale, s, which corresponds roughly to period (the reciprocal of frequency). Of particular importance to this study is the wavelet scale that corresponds to CF, termed $s_\heartsuit$ in this paper. The value of $s_\heartsuit$ is particular to the heart rate during a particular angiogram. Generally $s_\heartsuit$ is larger for humans than for piglets because the piglet heart rate is about 3 times faster than the human heart rate.

The complex continuous wavelet transform of a signal $f(t)$ is calculated by reference to a mother wavelet ψ, as given by the equation

$$W(u,s) = \frac{\int_{-\infty}^{\infty} f(t)\psi^{*}\left(\frac{t-u}{s}\right)\,dt}{\sqrt{s}}. \quad (1)$$

where s is wavelet scale, as above, u is the wavelet translation parameter, and the superscript * represents complex conjugation [26]. The mother wavelet ψ controls the temporal and frequency resolution of the wavelet transform. The wavelet cine imaging methods developed in this paper make use of wavelets of a range of frequency and temporal resolution. A mother wavelet family that offers an explicit balancing of frequency and temporal resolution is the Gabor (variably termed the Morlet) wavelet ψ, given in principle by the equation

$$\psi(t) = \frac{1}{\sqrt[4]{\pi}} e^{-\frac{t^2}{r^2}} e^{i\,t\,n} \quad (2)$$

where the parameters r and n mediate the relation between the Gaussian $e^{-\frac{t^2}{r^2}}$, which shapes the temporal resolution, and the complex sinusoid $e^{itn} = cos(t\,n) + i\,sin(t\,n)$, which gives the frequency specification [24, 25, 27–29]. The Gabor wavelet ψ family is used here to provide wavelet transforms of varying frequency and temporal resolution.

I employ over symbol notation in this paper to specify the frequency versus temporal resolution of a wavelet transform. A wavelet transform with unspecified resolution is denoted by the over bar symbol $\bar{}$ (for example, $\bar{c}_{i,j}$ is a wavelet transform of unspecified resolution of $c_{i,j}$, which represents the angiographic time intensity curve at the i,j^{th} pixel), a high temporal resolution wavelet transform is denoted by the over hat symbol $\hat{}$ (as in $\widehat{c}_{i,j}$), and a high frequency resolution wavelet transform is denoted by the over tilde symbol $\tilde{}$ (as in $\widetilde{c}_{i,j}$). Uniformly spaced time sampling is implied and the subscript t is generally omitted. Lower case symbols are used to denote pixel-wise time signals, in which case the pixel indices i,j may be implied. Upper case symbols are used to denote frame wise signals. Table XT3 in Supporting Information File SI-1 summarizes the mathematical notation used in this manuscript.

In this paper, filtering by CF wavelet scale $s_{\heartsuit}$ at inverse wavelet transformation is implied, where "filtering" means nullification of all wavelet coefficients other than those for $s_{\heartsuit}$ prior to inverse transformation.

The initial, naive, vascular PW cine imaging strategy in this paper was to reformat an angiogram from t frames of $n \times m$ pixels to an $n \times m$ array of signals each of length t, perform pixel-wise wavelet filtering for CF phenomena on each time signal, reformat back to t frames of $n \times m$ complex value data, and render each complex datum with the brightness-hue color model (Supporting Information File SI-1 Fig XF9).

In the notation of this paper, the naive strategy was to compute $\bar{c}_{i,j}$ for the time signal of each i,j^{th} pixel, filter for $s_{\heartsuit}$, inverse wavelet transform, and render. The Results section shows that this strategy proved unsuccessful, prompting the creation of numerically simulated angiographic data. The simulations, in turn, permitted the development of a reconstruction strategy based on the cross-correlation of wavelets of mixed high frequency and temporal resolution.

Computational Implementation Of Gabor Complex-Valued Wavelets

The wavelet library employed here (Mathematica 11, Wolfram Research, Urbana, Illinois, USA) was subjected to acceptance testing as described in Supporting Information File SI-1 Section 5.2. This wavelet computational library provides a function, $GaborWavelet[n]$, that reifies the Gabor wavelet ψ as described in Equation 2. The programmer chooses the value of the parameter n to control the comparative

temporal and frequency resolution of the wavelet transformation at computation. The acceptance testing led to the selection of the particular n parameter values of 1 for high temporal resolution wavelet transformation and equivalently 6 or 12 for high frequency wavelet transformation.

Two-Dimensional Simulated Optical Angiography

The initial wavelet strategy for PW imaging proved unsatisfactory due to overwhelming artifact. The findings are shown in the Results section below. To develop a refined method of wavelet PW imaging, I developed a system for generating numerically simulated optical angiograms. The idea is to generate optical angiograms with known pulse motion and vascular pulsation to see if a given wavelet reconstruction method can recover the correct result.

A still NIR human image is obtained by averaging 300 NIR image frames of an example NIR video. To simulate tissue motion, the still image is computationally translated to and fro with bilinear 2-D interpolation according to the two-dimensional motions measured from the visible video. This is illustrated in exaggerated form by the Supporting Information Video File SI-41. To simulate CF flow variation the intensity of each pixel is varied at two summed frequency components: the frequency of one event per sampling interval where the signal intensity is low at the start and end of the sampling interval and reaches a peak at the midpoint, to simulate bolus passage, and at CF. Pulse motion and flow variation are variably mixed into the same simulation. The video files for the simulated angiograms are supplied as Supporting Information Videos SI-41 through SI-53.

One-Dimensional Simulated Optical Angiography

In an angiogram, a given pixel may be taken to represent a one-dimensional angiographic time intensity curve. To pursue wavelet reconstruction methods at the level of a pixel-wise time intensity curve, I generated simulated angiographic time intensity curves for analysis. These one-dimensional signals variably contain a synthetic bolus passage, CF variation, and random noise. They are subjected to wavelet transformation with wavelet ψs of varying temporal and frequency resolution, and the result is compared to the known properties of the synthetic signal.

Angiographic Time of Flight and Arteriovenous Pixel Classification

ATOF is used to classify pixels as arterial versus venous. I evaluated several methods for estimating ATOF to a pixel including time to peak signal value, mean arrival time, and the phase of the frequency one Fourier component of the pixel's circulatory time signal. The frequency one component of the Fourier transform has properties that are helpful in the modeling of a bolus passage. Like a bolus passage, it is a sinusoid event that occurs once per sampling interval. It has some advantages over other parameters such as the mean arrival time. For example, it filters out signals at other frequencies that do not correspond to the bolus passage, such as respiratory frequency and CF phenomena. Analysis with these methods of estimating ATOF reveals them to perform roughly equivalently with the human data but not with the piglet data. Testing shows the most robust results with the piglet data to be yielded by the phase of the Fourier frequency one component, so this method was used for both the human and piglet data.

The magnitude of the Fourier frequency one component relates to the areas under the time intensity curve, providing an analogy to the area under the curve in tracer kinetic methods [30, 31]. To enhance comparisons between subjects and species in these

data, bolus passage time is normalized by heart rate such that for a given subject it ranges from 0 to the number of heart beats recorded in a sampling interval. Since the angiographic images are obtained at faster than cardiac rate, ATOF is measured here with fractional heart beat precision.

Time-Indexed Phase Histogram

The distribution of PW phase in a given time slice may be analyzed by a phase histogram. The comparative distributions of arterial and venous phase in a time slice may be analyzed by comparing phase histograms of each. The temporal progression of arterial and venous phase may be analyzed by constructing a phase histogram of each for each time slice followed by aligning these in sequence to produce a time-indexed phase histogram. A time-indexed phase histogram has three dimensions. The horizontal axis is time, expressed as heartbeats to facilitate comparisons between the human and piglet data. The vertical axis is PW phase, ranging as $[-\pi, \pi]$. The third dimension is relative histogram count, reflected in pixel brightness, with arterial phase counts in red and venous in blue. Fig XF18 of Supporting Information File SI-1 illustrates the steps in generating a time-indexed phase histogram with arteriovenous classification.

Results

The Methods section above describes the physical data acquisition and the baseline computational methods for PW imaging. Since the initial results contain dense artifact, the computational methods were refined to offer interpretable results. The initial results, the refinement of the computational methods, and the yielded results are described in this section.

Angiographic and Motion Data

The figures in this document for the human and piglet data are based on subjects h1 and p1 (summarized in Fig 2). The figures for the other subjects are in Supporting Information File SI-1. The video files that comprise this paper's raw data and video renderings are provided as Supporting Information Videos and their file names are listed in Supporting Information File SI-1 Table XT4. The aneurysm location, heart rate, and signal to noise ratios (SNRs) for the CF component of the bolus passage and brain pulse motion are given in Supporting Information File SI-1 Tables XT1 and XT2 for the human and piglet data respectively.

Naive Wavelet Filter

The filter method proved unsatisfactory for the human optical data because the rendered images have excess artifact that appears as phase bimodality that is oriented longitudinally along vessels (Fig 3). The wavelet reconstructions offered by this method are attached as Supporting Information Video Files, with their file names given by the column headed by the symbol $\tilde{c}$ in Table XT5 of Supporting Information File SI-1. An explanation is that there is CF pulse tissue motion perpendicular to the course of the vessel that causes the vessel to shift side to side at CF. This causes contrast in the vessel to shift to and from adjacent regions perpendicular to the course of the vessel at CF, producing the longitudinal bimodality, a putative artifact termed here motion alias.

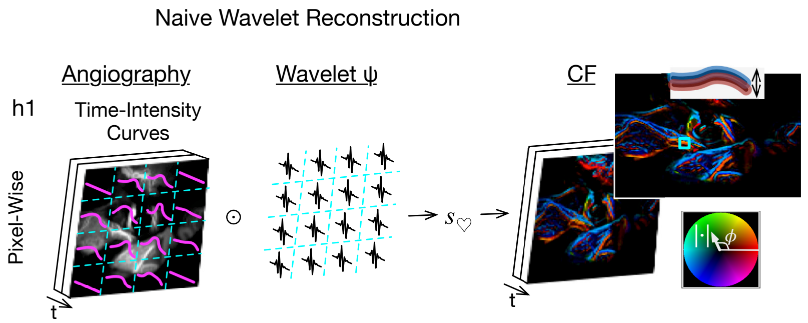

Fig 3. Naive Wavelet Angiography And Motion Alias. Apply wavelet transforms (⊙) to the pixel-wise time signals, filter for cardiac wavelet scale ($s_\heartsuit$), and render by a brightness-hue color model that represents CF magnitude as brightness and phase as hue (right bottom inset). The double inset top right shows motion alias in the lengthwise bimodal phase.

Simulated Optical Angiography

The simulated optical angiograms are filtered for CF angiographic phenomena using wavelet ψs of varying frequency and temporal resolution. Some two-dimensional simulated optical angiograms and their wavelet reconstructions are attached as Supporting Information Video Files, with the file names listed in Table XT4 of Supporting Information File SI-1. Inspection of the results from the naive CF wavelet filter as applied to the simulated angiograms discloses that:

1. If there is CF pulse motion but no flow variation, then the result has motion alias, regardless of the wavelet ψ (first row of Fig 4).

2. If there is CF flow variation but no motion, then the result has no motion alias, regardless of the wavelet ψ (second row of Fig 4).

3. If there is both CF motion and flow variation, then there is a proportionate mixture of these where a high frequency resolution reconstruction does but high temporal resolution does not have motion alias.

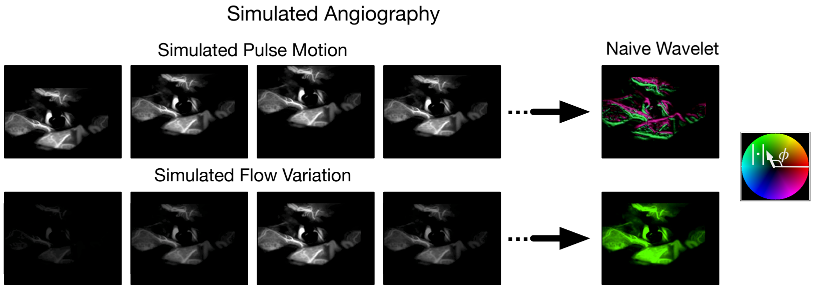

Fig 4. Optical Angiogram Simulation. The top row is simulated pulse motion. The bottom row is simulated intraluminal contrast variation. The right column shows an image after naive CF wavelet filtering. The simulated pulse motion and intraluminal contrast variation can be combined.

Since the longitudinal bimodality can be reproduced in naive wavelet reconstructions of simulated angiograms with high proportions of pulse motion, the longitudinal

bimodality of phase must reflect motion alias. Moreover, the results show that motion alias can be mitigated by filtering with a high temporal resolution ψ.

However, reliance on a high temporal resolution ψ may produce other facts due to the loss of frequency resolution. Testing against simulated one-dimensional data representative of a pixel-wise time signal shows that a naive CF wavelet filter based on a high temporal wavelet transform lets non-CF phenomena pass through, giving a separate type of artifact, frequency alias (Supporting Information File SI-1 Fig XF12).

Further testing with one-dimensional simulated data shows that motion and frequency alias may jointly be mitigated by the cross-correlation of high temporal wavelet transforms of the pixel-wise time signals by a high frequency resolution wavelet transform of the frame wise time intensity curve (giving $\widetilde{C}$, where the subscript t is implied). Please see Supporting Information File SI-1 Section 5.3.3 particularly Fig XF13 for details). The cross-correlation is performed by element wise multiplication after wavelet transformation [32–34].

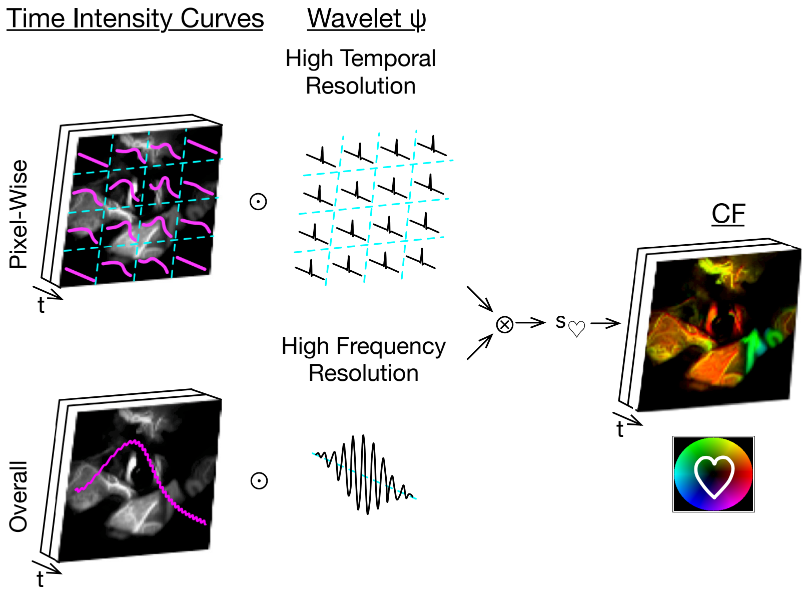

Fig 5. Cross-Correlated Wavelet Angiography. Apply high temporal resolution wavelet ($\odot$) transformation to the pixel-wise time intensity curves. Apply high frequency resolution wavelet transformation to the overall time intensity curve. Cross-correlate these pixel-wise ($\otimes$), filter for cardiac wavelet scale ($s_{\heartsuit}$), inverse wavelet transform, then cine render.

In the notation of this paper, the proposed spatiotemporal CF filter by wavelet cross-correlation is then

$$\widehat{x} = \widehat{c}\,\widetilde{C}^{*}. \tag{3}$$

The time domain VPW cine images are obtained by inverse wavelet transformation of $\widehat{x}$ with filtering by $s_{\heartsuit}$ (for details see the Materials and Methods section).

Mitigation of Artifact Summary

The two-dimensional and one-dimensional optical angiograms simulations suggest the following strategy for mitigating artifact at wavelet angiography:

1. Employ a high temporal resolution wavelet ψ for the pixel-wise circulatory signal transforms $\widehat{c}_{i,j}$ to mitigate motion alias.
2. Apply a high frequency resolution wavelet ψ transform to the overall circulatory signal normalized as $\frac{\widetilde{C}}{|\widetilde{C}|}$.
3. Perform pixel-wise cross-correlation as $\widehat{x}_{t,i,j} = \widehat{c}_{t,i,j} \frac{\widetilde{C}_t^*}{|\widetilde{C}_t|}$ to restore the loss of frequency resolution from the pixel-wise high temporal resolution ψ transform, with a possible second order correction to counter residual low frequency alias from the bolus passage.
4. Apply mean signal norming as point-wise multiplication along t of $x_{i,j}$ by C_t.

Please see Supporting Information File SI-1 Sections 5.3 and 5.4 for further details on these artifacts and their mitigation.

Reference to Pulse Motion

Having produced a computational wavelet system for cine imaging vascular PW that mitigates motion and frequency alias, we turn attention to developing a way of applying motion referencing to cine images of vascular PWs. The definition of $\widehat{x}$ may be adjusted to include reference to CF pulse motion by adjusting with complex-valued arithmetic the high frequency correland $\widetilde{C}$ in Equation 4 by the phase of pulse motion, $\widetilde{M}$, to give $\widetilde{C}\widetilde{M}^*$. Substituting $\widetilde{C}\widetilde{M}^*$for $\widetilde{C}$ in Equation 3 gives

$$\widehat{y} = \widehat{c}(\widetilde{C}\widetilde{M}^*)^* = \widehat{c}\widetilde{C}^*\widetilde{M}. \tag{4}$$

The motion-referenced spatiotemporal decompositions given by $\widehat{y}$ may be obtained as above by inverse wavelet transformation with filtering by $s_\heartsuit$ and rendering as video files (see the file names in the last column of Table XT4). Perusal of these video files shows the motion-referenced vascular PW cine images to look similar to the non-motion-referenced ones except that there is creative constancy of phase (rendered as color) across each heart beat. Time-indexed phase histograms of motion-referenced phase are constructed as for the time-indexed phase histograms above.

Human and Piglet Vascular Pulse Wave Imaging

Having refined the wavelet methods, we return to their application to the human and piglet data.

The vascular PW cine images may be rendered with the brightness-hue model as video files for inspection. The video files have three dimensions, two spatial and one temporal. The raw data video files and their wavelet PW reconstructions are described in Supporting Information File 1 Table XT1 and are provided as Supporting Information Videos SI-3 through SI-40. For representation on a printed page a two dimensional spatial snapshot may be selected from a given time coordinate. Selected snapshots of $\widehat{x}$ for h1 and p1 are presented in Fig 6 and for the other subjects in Supporting Information File SI-1 Fig XF15.

Angiographic Time of Flight and Arteriovenous Pixel Classification

Histograms of ATOF generally approximate a bimodal shape (Figs 7 and SI-1-XF16). There is extended venous stasis and evidence of angiographic recirculation in some of the subjects that impairs the estimation of the mean angiographic transit time, but

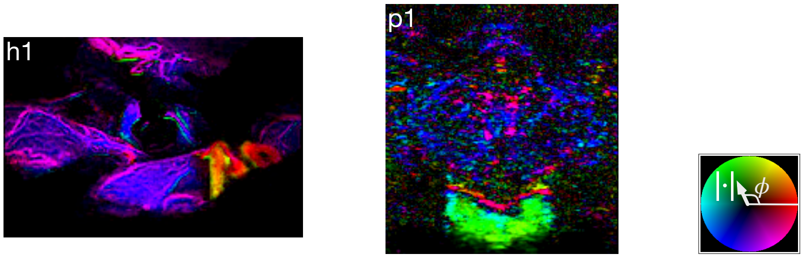

Fig 6. Wavelet Angiography Snapshots.

inspection of the ATOF histograms suggests it to be in the range of 10 to 40 heartbeats across the subjects. For each subject, the ATOF value of the deepest valley was selected as the arterial versus venous classification threshold. For the humans, those vessels classified as arterial versus venous corresponds to the impression based on visual inspection of the actual surgical field and the visible video. For the piglets, the classification of major arteries and veins corresponds roughly to a piglet atlas (Fig SI-1-XF6). There must be some statistical error in the binary classification of pixels as arterial versus venous. Nonetheless, the time intensity curves drawn from pixels classified as arterial and venous have shapes consistent respectively with the arterial and venous circulations because the time intensity curves drawn from pixels classified as arterial peak earlier than the time intensity curves from pixels classified as venous.

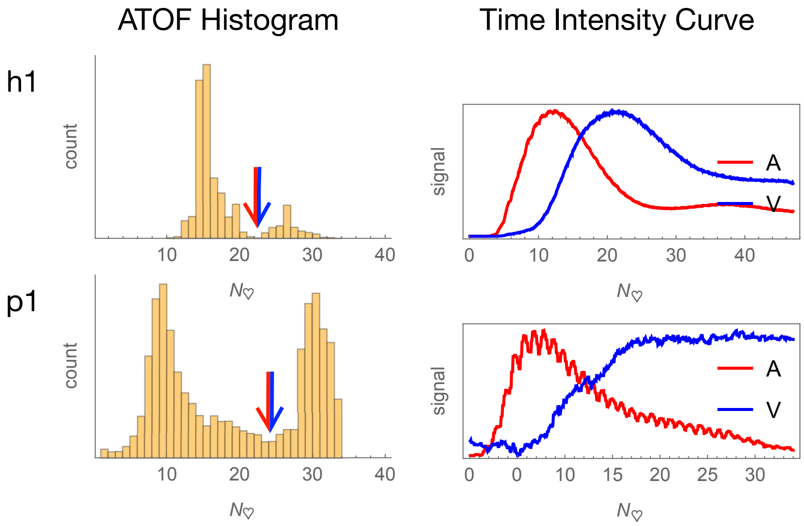

Fig 7. Arteriovenous Classification. Arterial and venous pixels are separated according to angiographic time of flight (ATOF). The derived time intensity curves are respectively consistent with predominantly arterial and venous temporal behavior.

Arterial and Venous Time-Indexed Phase Histograms

The time-indexed phase histograms for subjects h1 and p1 are shown in Fig 8 and for the other subjects in Supporting Information File SI-1 Fig XF19. Consistent with the ATOF criteria employed to classify pixels as arterial versus venous, red (arterial) comparatively predominates on the left early in the bolus passage and blue (venous) on the right.

Visual inspection of the merged arterial and venous time-indexed phase histograms

appears to show the presence of a consistent arterial versus venous phase difference across the bolus passage. Line scans are drawn through each of the time-indexed-phase histograms to depict further detail on arterial and venous phase (second columns of Figs 8 and SI-1-XF19). These data may be interpreted as showing the presence of resolvable arterial and venous pulsations in both human and piglet brain.

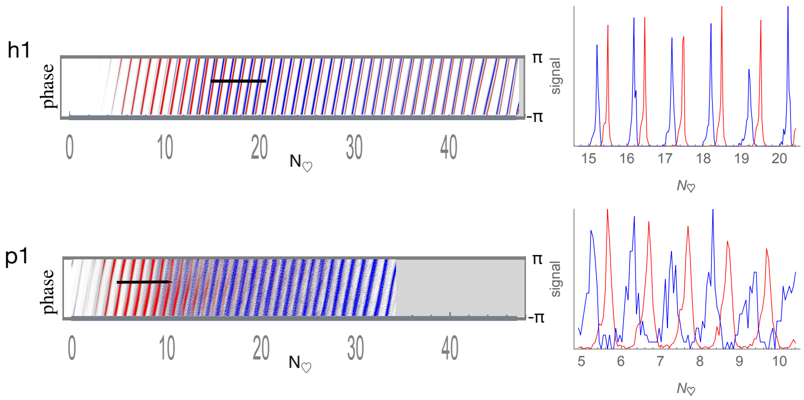

Fig 8. Arterial And Venous Time-Indexed Phase Histograms With Line Scans. Pixels classified by ATOF as arterial are in red and those as venous in blue. Relative histogram count is represented as brightness. The horizontal axis is time is depicted in heartbeats to facilitate human-piglet comparison. The vertical axis is phase ranging as $[-\pi, \pi]$. There is a consistent arteriovenous phase difference across the bolus passage.

Arterial and Venous Phase Histograms after Reference to Brain Pulse Motion

The phase histograms may be computed from the motion-referenced wavelet angiograms to show the relationship between angiographic PW phase and pulse motion phase. Fig 9 shows the motion-referenced time-indexed phase histograms for subjects h1 and p1. The other subjects are shown in Supporting Information File SI-1 Fig XF20. The arterial and venous phases maintain a relatively constant phase difference across the bolus passage consistent with an arteriovenous phase lock. When the motion-referenced phases are summed across the time of the bolus passage and represented as circular histograms, there remains an overall arteriovenous PW phase difference (right column of Figs 9 and SI-1-XF20).

Discussion

This paper reports exploratory vascular PW cine imaging by a wavelet angiography method capable of resolving individual PWs, and finds arterial and venous PWs to have separate phase relations to each other and to brain pulse motion in both humans and piglets (Figs 8 and 9). The arteriovenous phase lock is consistent with the hypothesis that there is information exchange between the arterial and venous components of cerebral circulation. This phase lock may be an experimental signature of a coordinating mechanism whereby an arriving stroke volume of arterial blood matches to an equivalent ejection of venous blood.

These exploratory data furthermore suggest the presence of a trilateral phase relationship relationship between the phases of arterial PWs, venous PWs, and brain pulse motion. A rudimentary interpretation of these data is that arterial wall expansion

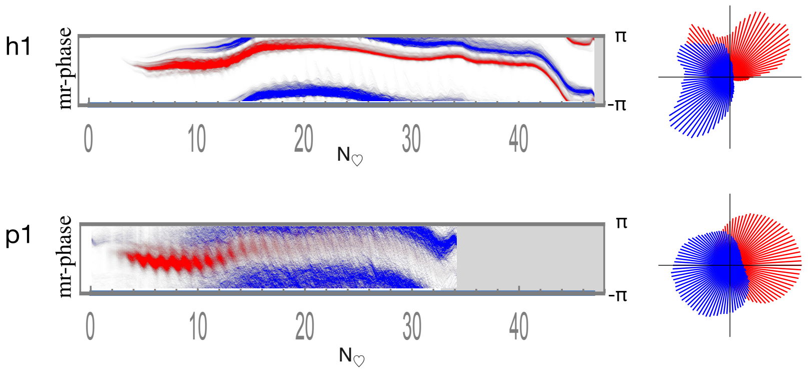

Fig 9. Arterial And Venous Motion-Referenced Phase Histograms. Pixels classified by ATOF as arterial are in red and those as venous in blue. Relative histogram count is represented as brightness. The horizontal axis is time is depicted in heartbeats to facilitate human-piglet comparison. The vertical axis is phase ranging as $[-\pi, \pi]$. Arterial versus venous phase appears approximately fixed in difference across the entire bolus passage. The circular phase histograms on the right are calculated by summing the time-indexed phase histograms on the left across time.

displaces extravascular tissues to produce venous wall compression by local conservation of mass. Even such a simple model implies complex topological arrangements in tissue between arterial and venous vasculature and places constraints on the biophysical properties of the intervening brain tissue [35]. The presence of these implied properties may be subject to experimental testing. These data permit no comment on whether the brain has an active regulatory system with PW sensors integrated to vascular effectors that can modulate spatiotemporal PW phenomena. The potential role of alterations in biomechanical tissue properties on arteriovenous PW interactions is a topic for further research.

I do not report data outside of the brain but it is possible that parallel arteriovenous information exchange occurs throughout the entire central nervous system including within the intradural spinal component [36].

Limitations

Of the four human subject set aside for wavelet analysis, the features of their optical angiograms that make them suitable for wavelet analysis (no motion of the microscope, no manipulation of the vessels, and inclusion of the venous phase of the angiogram) seem unlikely to have influence that distribution of vascular PWs and of brain pulse motion. Therefore I assess as unlikely that the core findings reported here are unduly influenced by a selection factor.

The geometry of human brain surface optical angiography proves well suited to the detection of motion alias because the exposed brain surface vessels are perpendicular to the line of sight of the operating microscope. The same surface geometry simplifies the numerical simulation of angiography. The angiographic simulation could be improved by the incorporation of other signal sources such as respiratory frequency phenomena.

By contrast to optical brain surface angiography, the cross-sectional geometry of the piglet cranial window ultrasound angiography method does not lend itself as easily neither to the detection of angiographic motion alias nor to the generation of simulated angiography, since such would require perhaps a three dimensional simulation. Nonetheless, the measurability of pulse motion in the raw cine data is consistent with the presence of motion alias and justifies the use of the cross-correlated wavelet method.

Neither of these two angiographic methods captures three dimensional spatial data. The suggestion here of arterial and venous PW complementarity is based on the observation of arteriovenous phase locking. Were a three dimensional wavelet angiography method to be employed, one would experimentally predict the the presence of CF oscillating arterial and venous blood volumes that sum to an approximate constant in the central nervous system throughout the cardiac cycle [37].

This cross-correlated wavelet method attenuates both motion and frequency alias sufficient for testing the hypothesis of this paper, but the region of interest for pulse motion tracking is selected opportunistically by visual inspection. The location of the region of motion tracking interest influences the individual phase relationships between pulse motion and arterial and venous PWs but it does not impact the measured phase differences between arterial and venous PWs.

Since the brain surface optical technique is performed at open cerebrovascular craniotomy, there is no intact cranial vault to constrain brain pulse motions. This factor limits the generalizability of these human data since pulse motion may be of lower magnitude when the skull is intact. If further research were to establish that there is less pulse brain motion in human high speed angiographic data acquired with a closed cranium, it might be feasible to mitigate motion alias with a wavelet ψ of less high temporal resolution than employed here, thereby mitigating the frequency alias that comes with the use of an extreme high temporal resolution wavelet ψ.

The ICP Waveform

The management of ICP via implanted devices plays a role in the care of patients with closed head injury, spontaneous intracerebral hemorrhage, and cerebral edema [13, 38]. There is CF variation to ICP but the basis of the ICP waveform remains unknown [39, 40]. The addition of a method such as reported here for vascular PW cine imaging may enable an experimental strategy for reconstructing the causal chain that produces the ICP waveform.

The Brain Ventricles

The relationships between brain pulse motion and arterial and venous PWs reported here raise the question of whether an arterial PW may cause coordinated pulse motions of brain ventricle walls [41]. The low viscosity and lack of internal structure to CSF may enable the ventricles to provide a low friction relay medium for PW coupling, possibly extending the spatiotemporal window for effective coupling. This formulation experimentally predicts an association between vascular PWs about the surfaces of the ventricles, ventricle walls motions, and adjacent internal CSF pressure variations.

Selected human patients with hydrocephalus who undergo attempted endoscopic treatment offer a brief opportunity for the internal direct observation of ventricle wall motions and adjacent CSF pressure variations [42]. In a companion paper, my coauthors and I report direct intraoperative observations from within the lateral and third ventricles of three such human subjects and we compare the measurements to the distribution in piglets of vascular PWs over the ventricular surfaces (companion manuscript attached here as Supporting Information SI-55).

These and further studies may shed light on whether the ventricles serve to shape a low viscosity medium, CSF, so as to enable vascular PW coupling across remote vascular distributions. The ventricles remain the largest structure in the human body without established primary purpose, even though their existence has been known at least since 350 BCE when Aristotle observed of the brain "in the great majority of animals it has a small hollow in its centre" [43].

Supporting Information

SI-1 File. Supporting Information document on methods and results for human and piglet wavelet angiography, mathematical methods and numerical angiography simulation. Contains supporting text, figures, and tables.

SI-2 Video File. Visible brain surface video for human subject h1.

SI-3 Video File. Optical brain surface angiography video for human subject h1.

SI-4 Video File. High temporal resolution CF wavelet filter of SI-4.mov.

SI-5 Video File. High frequency resolution CF wavelet filter of SI-4.mov.

SI-6 Video File. Cross-correlated wavelet CF wavelet filter of SI-4.mov.

SI-7 Video File. Motion-referenced cross-correlated wavelet CF wavelet filter of SI-4.mov.

SI-8 Video File. Visible brain surface video for human subject h2.

SI-9 Video File. Optical brain surface angiography video for human subject h2.

SI-10 Video File. High temporal resolution CF wavelet filter of SI-10.mov.

SI-11 Video File. High frequency resolution CF wavelet filter of SI-10.mov.

SI-12 Video File. Cross-correlated wavelet CF wavelet filter of SI-10.mov.

SI-13 Video File. Motion-referenced cross-correlated wavelet CF wavelet filter of SI-10.mov.

SI-14 Video File. Visible brain surface video for human subject h3.

SI-15 Video File. Optical brain surface angiography video for human subject h3.

SI-16 Video File. High temporal resolution CF wavelet filter of SI-16.mov.

SI-17 Video File. High frequency resolution CF wavelet filter of SI-16.mov.

SI-18 Video File. Cross-correlated wavelet CF wavelet filter of SI-16.mov.

SI-19 Video File. Motion-referenced cross-correlated wavelet CF wavelet filter of SI-16.mov.

SI-20 Video File. Visible brain surface video for human subject h4.

SI-21 Video File. Optical brain surface angiography video for human subject h4.

SI-22 Video File. High temporal resolution CF wavelet filter of SI-22.mov.

SI-23 Video File. High frequency resolution CF wavelet filter of SI-22.mov.

SI-24 Video File. Cross-correlated wavelet CF wavelet filter of SI-22.mov.

SI-25 Video File. Motion-referenced cross-correlated wavelet CF wavelet filter of SI-22.mov.

SI-26 Video File. Cranial window ultrasound angiography video for piglet subject p1.

SI-27 Video File. High temporal resolution CF wavelet filter of SI-27.mov.

SI-28 Video File. High frequency resolution CF wavelet filter of SI-27.mov.

SI-29 Video File. Cross-correlated wavelet CF wavelet filter of SI-27.mov.

SI-30 Video File. Motion-referenced cross-correlated wavelet CF wavelet filter of SI-27.mov.

SI-31 Video File. Cranial window ultrasound angiography video for piglet subject p2.

SI-32 Video File. High temporal resolution CF wavelet filter of SI-32.mov.

SI-33 Video File. High frequency resolution CF wavelet filter of SI-32.mov.

SI-34 Video File. Cross-correlated wavelet CF wavelet filter of SI-32.mov.

SI-35 Video File. Motion-referenced cross-correlated wavelet CF wavelet filter of SI-32.mov.

SI-36 Video File. Cranial window ultrasound angiography video for piglet subject p3.

SI-37 Video File. High temporal resolution CF wavelet filter of SI-36.mov.

SI-38 Video File. High frequency resolution CF wavelet filter of SI-37.mov.

SI-39 Video File. Cross-correlated wavelet CF wavelet filter of SI-37.mov.

SI-40 Video File. Motion-referenced cross-correlated wavelet CF wavelet filter of SI-37.mov.

SI-41 Video File. Simulated human brain surface optical angiogram with exaggerated pulse motion.

SI-42 Video File. Simulated human brain surface optical angiogram with pulse motion.

SI-43 Video File. High temporal resolution CF wavelet filter of simulated human brain surface optical angiogram with pulse motion.

SI-44 Video File. High frequency resolution CF wavelet filter of simulated human brain surface optical angiogram with pulse motion.

SI-45 Video File. Cross-correlated CF wavelet filter of simulated human brain surface optical angiogram with pulse motion.

SI-46 Video File. Simulated human brain surface optical angiogram with pulse flow.

SI-47 Video File. High temporal resolution CF wavelet filter of simulated human brain surface optical angiogram with pulse flow.

SI-48 Video File. High frequency resolution CF wavelet filter of simulated human brain surface optical angiogram with pulse flow.

SI-49 Video File. Cross-correlated CF wavelet filter of simulated human brain surface optical angiogram with pulse flow.

SI-50 Video File. Simulated human brain surface optical angiogram with both pulse motion and pulse flow.

SI-51 Video File. High temporal resolution CF wavelet filter of simulated human brain surface optical angiogram with both pulse motion and pulse flow.

SI-52 Video File. High frequency resolution CF wavelet filter of simulated human brain surface optical angiogram with both pulse motion and pulse flow.

SI-53 Video File. Cross-correlated CF wavelet filter of simulated human brain surface optical angiogram with both pulse motion and pulse flow.

SI-54 Video File. Visible-NIR Video Alignment.Demo of graphical user interface program for synchronizing human visible and NIR videos.

SI-55 File. CSF in the Ventricles of the Brain Behaves as a Relay Medium for Arteriovenous Pulse Wave Coupling (companion manuscript).

Acknowledgments

Benjamin I. Rapoport contributed an early Fourier-based version of the pulsation image phase reconstruction algorithm that inspired the development of the wavelet-based one. In addition, he contributed the brightness-hue model for rendering complex-valued data. Saad Khan helped create and implement the piglet cranial window ultrasound model. Pankaj K. Agarwalla, Paul H. Chapman, Patricia K. Donahoe, Ann Christine Duhaime, Bernard Kinane, Elizabeth G. Shannon, Ralph Weissleder and Ziv Williams commented on versions of the manuscript. There was an earlier version of the manuscript that contained only the piglet data. James Baish and Lance Munn provided criticisms of it that prompted me to gather the human data and redirect the argument. The piglet cranial window studies were funded by Center for Integration of Medicine and Innovative Technology (CIMIT) grant a0K300000022wE4000840.

References

1. Wilson MH. Monro-Kellie 2.0: The dynamic vascular and venous pathophysiological components of intracranial pressure. Journal of Cerebral Blood Flow & Metabolism. 2016;36(8):1338–1350.

2. Tomita M, Osada T, Schiszler I, Tomita Y, Unekawa M, Toriumi H, et al. Automated method for tracking vast numbers of FITC-labeled RBCs in microvessels of rat brain in vivo using a high-speed confocal microscope system. Microcirculation (New York, NY: 1994). 2008;15(2):163–174.

3. Nyqvist H. Certain topics in telegraph transmission theory. Transactions of the AIEE. 1928;47:617–644.

4. Shannon CE. Communication in the Presence of Noise. Proceedings of the Institute of Radio Engineers. 1949;37(1):10–21.

5. Kotelnikov VA. On the capacity of the ‘ether’ and of cables in electrical communication. In: Procs. of the first All-Union Conference on the technological reconstruction of the communications sector and low-current engineering. Moscow; 1933.

6. Unser M. Sampling—50 Years After Shannon. Proceedings of the IEEE. 2000;88(4):569–587.

7. Bhadelia RA, Bogdan AR, Kaplan RF, Wolpert SM. Cerebrospinal fluid pulsation amplitude and its quantitative relationship to cerebral blood flow pulsations: a phase-contrast MR flow imaging study. Neuroradiology. 1997;39(4):258–264.

8. Kim MH, Shin KM, Song JH. Cine MR CSF flow study in hydrocephalus: what are the valuable parameters? Acta neurochirurgica Supplement. 1998;71(6):343–346.

9. Markl M, Frydrychowicz A, Kozerke S, Hope M, Wieben O. 4D flow MRI. Journal of Magnetic Resonance Imaging. 2012;36(5):1015–1036.

10. Kachelriess M, Ulzheimer S, Kalender WA. ECG-correlated image reconstruction from subsecond multi-slice spiral CT scans of the heart. Medical Physics. 2000;27(8):1881–1902.

11. Bernstein MA, King KF, Zhou XJ. Handbook of MRI Pulse Sequences. Elsevier; 2004.

12. Kim DJ, Czosnyka M, Kim H, Balédent O, Smielewski P, Garnett MR, et al. Phase-shift between arterial flow and ICP pulse during infusion test. Acta Neurochirurgica. 2015;157(4):633–638.

13. Kawoos U, McCarron RM, Auker CR, Chavko M. Advances in Intracranial Pressure Monitoring and Its Significance in Managing Traumatic Brain Injury. International Journal of Molecular Sciences. 2015;16(12):28979–28997.

14. Wagshul ME, Eide PK, Madsen JR. The pulsating brain: A review of experimental and clinical studies of intracranial pulsatility. Fluids Barriers CNS. 2011;8(1):5.

15. Lawton MT. Seven Aneurysms: Tenets and Techniques for Clipping. New York: Thieme; 2011.

16. Zaidi HA, Abla AA, Nakaji P, Chowdhry SA, Albuquerque FC, Spetzler RF. Indocyanine green angiography in the surgical management of cerebral arteriovenous malformations: lessons learned in 130 consecutive cases. Neurosurgery. 2014;10 Suppl 2:246–51– discussion 251.

17. Hardesty DA, Thind H, Zabramski JM, Spetzler RF, Nakaji P. Safety, efficacy, and cost of intraoperative indocyanine green angiography compared to intraoperative catheter angiography in cerebral aneurysm surgery. Journal of clinical neuroscience: official journal of the Neurosurgical Society of Australasia. 2014;.

18. Hyvärinen L, Flower RW. Indocyanine green fluorescence angiography. Acta Ophthalmologica. 1980;58(4):528–538.

19. Raabe A, Nakaji P, Beck J, Kim LJ, Hsu FPK, Kamerman JD, et al. Prospective evaluation of surgical microscope-integrated intraoperative near-infrared indocyanine green videoangiography during aneurysm surgery. Journal of Neurosurgery. 2005;103(6):982–989.

20. Kuroiwa T, Kajimoto Y, Ohta T. Development and clinical application of near-infrared surgical microscope: preliminary report. Minimally Invasive Neurosurgery. 2001;44(4):240–242.

21. Aaslid R, Markwalder TM, Nornes H. Noninvasive transcranial Doppler ultrasound recording of flow velocity in basal cerebral arteries. Journal of Neurosurgery. 1982;57(6):769–774.

22. Meairs S, Alonso A. Ultrasound, microbubbles and the blood-brain barrier. Progress in Biophysics and Molecular Biology. 2007;93(1-3):354–362.

23. Daubechies I. The wavelet transform, time-frequency localization and signal analysis. IEEE Transactions on Information Theory. 1990;36(5):961–1005.

24. Gabor D. Theory of communication. Part 2: The analysis of hearing. Journal of the Institution of Electrical Engineers - Part III: Radio and Communication Engineering. 1946;93(26):442–445.

25. Goupillaud PL, Grossmann A, Morlet J. Cycle-octave and related transforms in seismic signal analysis. Geoexploration. 1984;23(1):85–102.

26. Daubechies I. 2. The Continuous Wavelet Transform. In: Ten Lectures on Wavelets. Society for Industrial and Applied Mathematics; 1992. p. 17–52.

27. Morlet J. Wave propagation and sampling theory—Part I: Complex signal and scattering in multilayered media. Geophysics. 1982;47(2):203.

28. Daubechies I. Ten Lectures on Wavelets. Society for Industrial and Applied Mathematics; 1992.

29. Ashmead J. Morlet Wavelets in Quantum Mechanics. Quanta. 2012;1(1):58–70.

30. Lassen NA, Perl W. Tracer Kinetic Methods in Medical Physiology. Raven, New York; 1979.

31. Hamberg LM, Hunter GJ, Halpern EF, Hoop B, Gazelle GS, Wolf GL. Quantitative high-resolution measurement of cerebrovascular physiology with slip-ring CT. American Journal of Neuroradiology. 1996;17(4):639–650.

32. Serroukh A. Wavelet coefficients cross-correlation analysis of time series. Electronic Journal of Applied Statistical Analysis. 2012;5(2):289–296.

33. Klimenko S, Mitselmakher G, Sazonov A. A cross-correlation technique in wavelet domain for detection of stochastic gravitational waves. General Relativity and Quantum Cosmology. 2002;.

34. Mizuno-Matsumoto Y, Ukai S, Ishii R, Date S, Kaishima T, Shinosaki K, et al. Wavelet-crosscorrelation analysis: Non-stationary analysis of neurophysiological signals. Brain Topography. 2005;17(4):237–252.

35. Goriely A, Geers MGD, Holzapfel GA, Jayamohan J, Jerusalem A, Sivaloganathan S, et al. Mechanics of the brain: perspectives, challenges, and opportunities. Biomechanics and modeling in mechanobiology. 2015;14(5):931–965.

36. Martin BA, Kalata W, Shaffer N, Fischer P, Luciano M, Loth F. Hydrodynamic and longitudinal impedance analysis of cerebrospinal fluid dynamics at the craniovertebral junction in type I Chiari malformation. PloS One. 2013;8(10):e75335.

37. Provost J, Papadacci C, Arango JE, Imbault M, Fink M, Gennisson JL, et al. 3D ultrafast ultrasound imaging in vivo. Physics in Medicine and Biology. 2014;59(19):L1.

38. Helbok R, Olson DM, Roux PDL, Vespa P, Monitoring TPitIMCCoM. Intracranial Pressure and Cerebral Perfusion Pressure Monitoring in Non-TBI Patients: Special Considerations. Neurocritical Care. 2014;21(2):85–94.

39. Balestreri M, Czosnyka M, Steiner LA, Schmidt E, Smielewski P, Matta B, et al. Intracranial hypertension: what additional information can be derived from ICP waveform after head injury? Acta Neurochirurgica. 2004;146(2):131–141.

40. Carrera E, Kim DJ, Castellani G, Zweifel C, Czosnyka Z, Kasprowicz M, et al. What Shapes Pulse Amplitude of Intracranial Pressure? Journal of Neurotrauma. 2009;27(2):317–324.

41. Zou R, Park EH, Kelly EM, Egnor M, Wagshul ME, Madsen JR. Intracranial pressure waves: characterization of a pulsation absorber with notch filter properties using systems analysis: laboratory investigation. Journal of Neurosurgery: Pediatrics. 2008;2(1):83–94.

42. Kulkarni AV, Drake JM, Mallucci CL, Sgouros S, Roth J, Constantini S, et al.;.

43. Aristotle. Historia Animalium. $http://classics.mit.edu/Aristotle/history_anim.mb.txt$; 350.
